# Temporal Trends, Multiple Residue Incidence, and Chronic Health Risk of Pesticides in Egyptian Onions: A Four-Year Market Surveillance

**DOI:** 10.3390/jox15060192

**Published:** 2025-11-10

**Authors:** Farag Malhat, Shokr Shokr, Sara Heikal, Nour El-Hoda Zidan

**Affiliations:** 1 Central Agricultural Pesticide Laboratory, Pesticide Residues and Environmental Pollution Department, Agricultural Research Center, Dokki, Giza 12618, Egypt; drshokr63@yahoo.com; 2 Central Agricultural Pesticide Laboratory, National Center for Monitoring Pesticide Residues and Pollutants, Agricultural Research Center, Dokki, Giza 12618, Egypt; 3 Pesticide Department, Faculty of Agriculture, Menofiya University, Shebien El-Kom 32514, Egypt; saraheikal2020@gmail.com; 4 Pesticides Chemistry and Toxicology Department, Faculty of Agriculture, Kafrelsheikh University, Kafr El-Sheikh 33516, Egypt; nourelhoda_az@hotmail.com

**Keywords:** *Allium cepa*, pesticide residues prevalence, MRL violations, multiple residues, chronic risk assessment

## Abstract

This study analyzed 5731 Egyptian onion samples collected from 2021 to 2024 to detect 430 pesticide residues and evaluate occurrence patterns, EU Maximum Residue Limits (MRLs) compliance, multiple residue prevalence, and consumer health implications. The analysis revealed temporal fluctuations in contamination, with residue-free samples ranging from 51.19% (2023) to 75.59% (2022) and MRL exceedances varying from 1.34% (2022) to 8.33% (2023). Throughout 2021–2024, fungicides dominated pesticide residues, declining from 70% to 50%, while insecticides increased from 30% to 40%. Pesticide detection patterns shifted significantly over the study period, with carbendazim decreasing from 20.99% to 2.35%, azoxystrobin fluctuating between 3.86% and 18.78%, and dimethomorph rising to 16.67%, reflecting evolving agricultural practices. Multiple residue occurrence fluctuated dramatically, from 9.76% (2022) to a peak of 30.82% (2023), while single residue occurrence remained more stable, ranging between 14.06% and 22.90%. Several pesticides exceeded EU MRLs, including imazalil, chlorpropham, chlorpyrifos, imidacloprid, and profenofos. Despite these violations, the EFSA PRIMo 3.1 model’s chronic risk assessment showed that exposure levels for all population groups remained well below Acceptable Daily Intake (ADI) thresholds, with a maximum %ADI of 2.27% for chlorpyrifos in high-consumption regions.

## 1. Introduction

The onion (*Allium cepa* L.), a global staple cultivated worldwide as a profitable cash crop and indispensable culinary ingredient, is also recognized for its nutraceutical and medicinal properties [1]. Onions are nutrient-dense vegetables packed with essential vitamins, minerals, and bioactive compounds like quercetin and allicin that help to mitigate oxidative stress, reduce blood pressure, and offer anticancer and immune-boosting benefits [2]. In 2023, Egypt ranked third globally in onion production, with approximately 3.8 million tonnes and a gross production value of about USD 1.6 billion, while its onion exports totaled approximately 533,000 tonnes, valued at USD 207 million [3].

In Egypt, onion cultivation is significantly impacted by a range of pests and diseases that attack the crop both in the field and during storage. Key pests include onion thrips, aphids, and bulb flies, while diseases like downy mildew and purple blotch are prevalent during the growing season [4]. Additionally, post-harvest losses are often caused by various fungal and bacterial rots, such as neck rot, black mold, and bacterial soft rot, which thrive in storage conditions [5]. Consequently, the application of insecticides, fungicides, and bactericides is a necessary practice for farmers to protect yields and ensure the quality of the onion harvest.

However, pesticide residues can persist, leading to environmental contamination and health risks, highlighting the need for integrated pest management and residue monitoring. Monitoring pesticide levels in agricultural products fulfills several important purposes: ensuring regulatory compliance by quantifying residue levels on crops and verifying proper application practices; enhancing consumer safety and confidence; protecting environmental ecosystems; and facilitating international trade by meeting export standards [6,7].

Many researchers have examined the occurrence of pesticide residues in onions available in markets across the world, including those in China [8,9,10], the Republic of Korea [11], Iran [12], Vietnam [13], the Czech Republic [14], Spain [15], United Arab Emirates [16], Saudi Arabia [17], Bolivia [18], Ethiopia [19], Brazil [20], and the European Union [21].

Despite these valuable contributions, a comprehensive, multi-year market surveillance specifically focused on Egyptian onions, detailing temporal trends in pesticide residue levels, the incidence of multiple residues, and a thorough chronic health risk assessment tailored to this specific crop, remains underexplored, as previous Egyptian studies on pesticide residues in onions have several notable gaps. For instance, Malhat et al. [22,23] did not specify the seasonal distribution of their onion samples, which potentially limits the understanding of temporal variations in pesticide residue levels, while El-Sheikh et al. [24] restricted their analysis to a single governorate, thereby limiting external validity.

This study aimed to comprehensively monitor 430 pesticide residues in onions from Egyptian markets over a four-year period (2021–2024), evaluate compliance with EU MRLs, and assess potential health risks to consumers. The study sought to identify temporal trends in pesticide detection rates, determine the most frequently detected pesticide compounds and their concentration patterns, analyze the prevalence of single versus multiple residue contamination, and conduct chronic dietary risk assessments across diverse population groups using the EFSA PRIMo 3.1 model to establish whether current pesticide residue levels pose appreciable health risks to consumers through onion consumption.

## 2. Materials and Methods

### 2.1. Reagents, Chemicals, and Standards

The study employed a range of high-quality chemicals and tools sourced from reputable suppliers. Methanol (LC-grade) and acetonitrile (HPLC-grade) were supplied by Supelco (Merck), located in Darmstadt, Germany. Ammonium formate of analytical grade and formic acid of LC–MS grade, both possessing purities of 99% or greater and 98–100% respectively, were obtained from Sigma-Aldrich (Merck KGaA) in Darmstadt, Germany. QuEChERS kits, along with a salt extraction kit containing crucial components such as anhydrous magnesium sulfate and sodium chloride, were supplied by UCT, located in Bristol, PA, USA. Additionally, the dispersive solid-phase extraction kit from UCT contained specific quantities of primary–secondary amine (PSA) and magnesium sulfate. A collection of 430 individual high-purity pesticide standards (>99% purity) was sourced from Dr. Ehrenstorfer GmbH (Augsburg, Germany) and Sigma-Aldrich (Steinheim, Germany). The deionized water utilized in the experiments was generated through a Milli-Q water purification system.

### 2.2. Standard Solution Preparation

Standard stock solutions were formulated in toluene at a concentration of 1000 μg/mL and kept at −18 °C. From each of these solutions, a pesticide mixture was created, with each compound reaching a concentration of 10 μg/mL. Next, mixed standard working solutions at different concentrations were prepared by diluting the multi-analyte intermediate standard solution. The solutions were employed to prepare matrix-matched calibration standards and to effectively carry out tests for method recovery.

### 2.3. Gathering of Samples

From 2021 to 2024, inspectors collected a total of 5731 onion samples from various producers at wholesale markets in five Egyptian governorates (Gharbia, Qalyubia, Giza, Beheira, and El-Minofyia) to monitor for 430 multi-class pesticide residues. Adhering to the standardized sampling protocol outlined by the CODEX Alimentarius guidelines [25], these samples were collected randomly, and each weighed at least 3 kg. The samples were homogenized with a Hobart food processor model HCM62–1 (Hobart Corp., Troy, OH, USA) and stored in sealed, polypropylene containers in a refrigerator at 4 °C until analysis within approximately 24 h.

### 2.4. Preparation of Samples

Sample preparation was conducted according to the EN 15662:2018 [26] standard, using module E1 for extraction and module C2 for clean-up. A 10 g homogenized sample was placed in a 50 mL tube, and 10 mL of acetonitrile was subsequently added. The mixture was vigorously stirred for three minutes before adding the QuEChERS salt blend containing both anhydrous magnesium sulfate and sodium chloride to facilitate efficient phase separation. The sample was subsequently treated in a Geno/Grinder^®^ (SPEX SamplePrep, LLC, Metuchen, NJ, USA) for one minute and then centrifuged at 3000× *g* for five minutes.

During the clean-up, dispersive solid-phase extraction was utilized to eliminate interfering components from the sample matrix. A 1 mL portion of the crude extract was transferred into a 2 mL polypropylene minicentrifuge tube preloaded with 50 mg PSA and 300 mg anhydrous magnesium sulfate. The mixture was vortexed for one minute, followed by an additional five-minute centrifugation at 3000× *g*. The purified extract was then placed into a GC-MS/MS vial for analysis. A 1 mL sample of the unpurified acetonitrile extract was subjected to LC-MS/MS analysis without any prior cleanup steps.

### 2.5. Chromatographic Analysis

#### 2.5.1. LC-MS/MS Analysis

An AB SCIEX (USA) QTRAP 6500+ mass spectrometer, interfaced with a Shimadzu (Kyoto, Japan) LC-20 series HPLC system, was utilized for the analysis. A Phenomenex (Torrance, CA, USA) Synergi C18 reverse-phase column, kept at a temperature of 40 °C, was used to separate multi-residue compounds using gradient elution. The mobile phase system included Phase A, comprising 10 mM ammonium formate (pH 4) in a 90:10 water–methanol mixture (*v*/*v*), and Phase B, consisting of pure methanol, with a flow rate of 0.4 mL/min. The gradient elution program began with 100% Phase A for the first minute, followed by a linear decrease to 0% Phase A over 14 min. The system was then held at 0% Phase A for 3 min (15–18 min) to ensure full elution of late-eluting compounds, after which the composition was returned to 100% Phase A within 0.5 min and re-equilibrated for 2 min. The total run time was 20 min, with an injection volume of 2 µL.

Electrospray ionization (ESI) was performed using polarity-switching technology, allowing detection in both positive and negative ion modes with a 5.5 kV spray voltage. An ion source temperature of 400 °C was used, with curtain gas and collision gas both pressurized at 20 psi, while the nebulizer and auxiliary gas pressures were 35 psi. Multiple reaction monitoring (MRM) mode was employed in the mass spectrometer to monitor transitions from precursor ions to the two most abundant product ions for each analyte, in accordance with SANTE guidelines. Instrument control and data acquisition were managed using Analyst software (version 1.7.1) from AB SCIEX (Framingham, MA, USA). Retention times, quantification and confirmation MRM transitions, and collision energy parameters for LC compounds are listed in Table S1, while the associated total ion chromatograms (TICs) are shown in Figure S1.

#### 2.5.2. GC-MS/MS Analysis

Pesticides underwent thorough analysis utilizing a Thermo Scientific (Waltham, MA, USA) Trace 1300 gas chromatography system connected to a mass spectrometer equipped with an electron ionization source. An Agilent Technologies (Santa Clara, CA, USA) HP-5MS capillary column (30 m × 0.25 mm I.D., 0.25 µm film thickness) was employed for the separation. A 1.0 µL sample was injected using a split/splitless injector that was set to splitless mode. The programmed temperature for the PTV inlet began at 100 °C, held for 0.02 min, and then increased by 12 °C per second up to 320 °C, where it was maintained for 0.75 min. Helium was used as the carrier gas at a flow rate of 1.611 mL/min. The temperature program was: 80 °C, increased by 20 °C/min to 170 °C, then increased by 20 °C/min to 310 °C, with a final 3.5 min hold. The transfer line and ion source of the mass spectrometer were maintained at 250 °C and 300 °C, respectively. Compound detection was achieved using MRM mode, wherein two transitions were analyzed for each compound emerging from the GC (Table S2). All targeted pesticides were fully separated within the 15 min run time, evidenced by the TIC in Figure S2.

### 2.6. Method Validation

To validate the analytical method, we assessed key parameters—linearity, matrix effect, selectivity, sensitivity, accuracy, and precision—as required by the EU SANTE/11312/2021 guidelines [27]. To assess matrix effects, two sets of calibration curves were generated for both LC-MS/MS and GC-MS/MS analysis. One set was prepared in a pure solvent (acetonitrile), while the other was matrix-matched using an onion extract. For each curve, we used six concentration levels: 1, 5, 10, 25, 50, and 100 µg/L. A correlation coefficient of at least 0.99 was deemed acceptable for linearity.

The method’s selectivity was assessed by comparing chromatograms from solvent blanks and control onion samples against a pesticide mix standard, allowing us to differentiate analytes from background noise and other matrix components. The method’s sensitivity was assessed by determining the limit of quantification (LOQ) in spiked samples, which represents the minimum spiking concentration that satisfied the validation performance requirements.

We ensured acceptable accuracy, within the range of 70–120%, and precision, with a relative standard deviation (RSD) of no more than 20% (Table S3), by spiking blank samples with mixed standard solutions at concentrations of 0.005, 0.01, and 0.05 mg kg^−1^. Following this spiking, the samples were analyzed according to the method protocol, utilizing matrix-matched calibration curves for quantification. We assessed repeatability and within-laboratory reproducibility through multiple repetitions to calculate intra-day (*n* = 5) and inter-day (*n* = 15, over three consecutive days) RSD%. Lastly, we calculated the expanded measurement uncertainty (MU) using the reproducibility and trueness parameters, ensuring it remained within the guideline-specified threshold of 50% or below.

### 2.7. Quality Control and Quality Assurance

The analytical method for detecting multiple classes of pesticides in onions was validated at the Central Agricultural Pesticide Laboratory (CAPL) in Egypt, in accordance with the requirements of its ISO/IEC 17025:2017 [28] accreditation. The laboratory maintained data reliability by implementing a thorough quality assurance program that incorporated proficiency testing, spiked testing, intra-laboratory testing, and retention and replicate testing, all adhering to ISO/IEC 17025:2017 requirements.

### 2.8. Evaluation of Potential Health Hazards Associated with Onions Contaminated with Pesticides

To evaluate the long-term risks from pesticide residues in onions, the European Food Safety Authority’s (EFSA) Pesticide Residue Intake Model (EFSA PRIMo, revision 3.1) was employed [29]. The model integrates data from national food surveys carried out by EU Member States, encompassing information on onion intake and substantial portion sizes. The International Estimated Daily Intake (IEDI) is determined by taking into account different dietary patterns, the mean level of pesticide residues in onions, average body weight, and typical food consumption. To calculate chronic exposure as a percentage of the Acceptable Daily Intake (%ADI), the IEDI is divided by the ADI for the pesticides and then multiplied by 100. If this %ADI value surpasses 100%, it suggests that pesticide residues in onions could present a potential health risk to humans.

## 3. Results and Discussion

### 3.1. Determination of Pesticide Residues in Onion Samples and Evaluation of Adherence to EU MRLs

**Figure 1:**
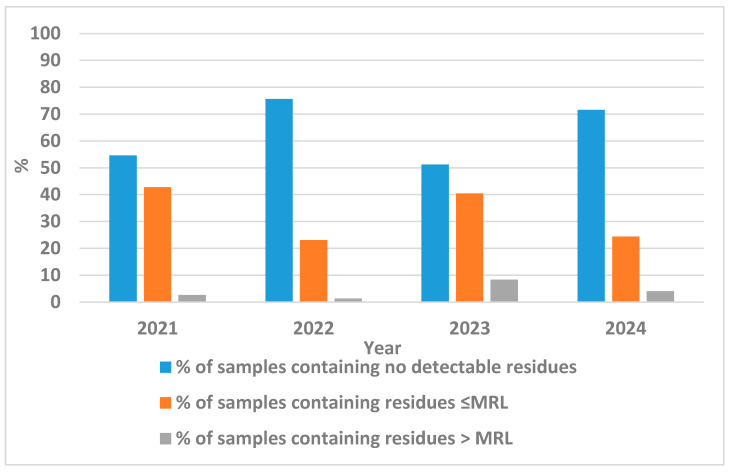
Temporal trends in pesticide residue detection and MRL compliance in Egyptian onions (2021–2024).

An examination of pesticide residue concentrations in onion samples obtained from markets in Egypt over the period from 2021 to 2024 reveals fluctuating trends in detection rates, compliance with EU MRLs, and exceedances, underscoring the dynamic interplay between agricultural practices, regulatory enforcement, and market surveillance (Figure 1). In 2021, with 262 samples analyzed, 32 distinct pesticides were detected, resulting in 54.58% of samples free of detectable residues, 42.75% complying with MRLs, and a relatively low 2.67% exceeding limits. The following year, 2022, saw a substantial increase in sample size to 2458, with 43 pesticides identified, yielding the highest proportion of residue-free samples at 75.59% and the lowest exceedance rate of 1.34%, alongside 23.07% within MRLs, which may reflect improved pest management. However, 2023 marked a concerning escalation, as 756 samples revealed 67 pesticides, the highest diversity observed, coupled with the lowest residue-free rate of 51.19%, 40.48% compliant with MRLs, and a peak exceedance of 8.33%, potentially attributable to intensified pesticide use amid pest pressures. By 2024, analysis of 2255 samples detected 54 pesticides, with 71.53% residue-free, 24.43% ≤ MRL, and 4.04% > MRL, indicating a partial recovery in compliance.

Residue-free rates in our study (51.19–75.59%) fall within the global range, which extends from 100% in Bolivian samples [18] to 0% in Vietnamese onions [13]. Other reported rates include 84.6% in the Republic of Korea [11], 65.8% in China [9], 88.3% in Brazil [20], and 25% in Saudi Arabia [17].

Our exceedance rates (1.34–8.33%) represent moderate levels when compared globally. While these rates are higher than the zero exceedances reported in the Republic of Korea [11], they are comparable to findings from other regions: 6% in China [9], 5.6% in United Arab Emirates (UAE) imports [16], 10% in Saudi Arabia [17], 8.2% in Brazil [20], and 11.7% in Egypt [22].

### 3.2. The Ten Pesticides Most Commonly Detected in Onion Samples

The frequency distribution of pesticide detections exhibited remarkable temporal shifts across the four-year study period, revealing dynamic changes in agricultural practices and regulatory responses in Egyptian onion production. Carbendazim demonstrated the most dramatic decline, plummeting from its peak detection frequency of 20.99% in 2021 to progressively lower levels of 10.09% in 2022, 5.56% in 2023, and ultimately 2.35% in 2024 (Table 1). Conversely, azoxystrobin maintained consistently high detection rates despite significant fluctuations, starting at 20.99% in 2021, and dropping to 3.86% in 2022, then resurging to become the most frequently detected pesticide at 18.78% in 2023 and 11.97% in 2024. Dimethomorph exhibited the most striking emergence pattern, transitioning from minimal detection frequencies of 2.67% in 2021 and 1.51% in 2022 to becoming the third most frequently detected pesticide at 16.67% in 2023 and 7.23% in 2024. Metalaxyl showed a persistent presence as the second most frequently detected pesticide with notable fluctuation from 10.69% in 2021 to 5.57% in 2022, peaking at 17.33% in 2023, and settling at 8.07% in 2024. Several pesticides demonstrated sporadic appearance patterns, with chlorpyrifos emerging prominently in 2022–2023 but disappearing from the top ten by 2024, while profenofos and lambda-cyhalothrin showed intermittent presence, and imidacloprid appeared only in the latter two years, collectively indicating shifting pesticide selection preferences and potentially evolving resistance management strategies among Egyptian onion producers.

The analysis revealed that mean concentrations of several pesticides exceeded EU MRLs across multiple years. In 2021, imazalil demonstrated the most severe violation with mean concentrations of 0.029 mg/kg, nearly three times the EU MRL of 0.01 mg/kg. The pattern of violations expanded in 2022, where chlorpropham exhibited mean concentrations of 0.014 mg/kg, exceeding its 0.01 mg/kg limit by 40%. In 2023, chlorpyrifos violations intensified with mean concentrations reaching 0.030 mg/kg, three times the acceptable limit, and imidacloprid joined the violators with mean residues of 0.015 mg/kg exceeding its 0.01 mg/kg MRL. By 2024, the violation pattern persisted with profenofos showing mean concentrations of 0.032 mg/kg against a 0.02 mg/kg limit, chlorpropham continuing its violations with mean residues of 0.021 mg/kg, and imidacloprid demonstrating the most severe violation with mean concentrations of 0.040 mg/kg, quadruple the EU MRL.

A comprehensive analysis of pesticide residues from 2021 to 2024 revealed fungicides as the most predominant class, constituting 70% of the most frequently detected pesticides in 2021 with a combined detection frequency of 65.57%, followed by 60% in both 2022 and 2023 with frequencies of 25.06% and 72.72% respectively, and 50% in 2024 at 35.70%; within this class, benzimidazoles like carbendazim and thiophanate-methyl were notable early on, with carbendazim decreasing from 20.99% in 2021 to 2.35% in 2024 and thiophanate-methyl detected only in 2022 at 2.24%, while strobilurins such as azoxystrobin fluctuated at 20.99% in 2021, 3.86% in 2022, 18.78% in 2023, and 11.97% in 2024; phenylamides like metalaxyl showed volatile frequencies of 10.69% in 2021, 5.57% in 2022, 17.33% in 2023, and 8.07% in 2024; morpholines, particularly dimethomorph, increased significantly from 2.67% in 2021 to 16.67% in 2023 and 7.23% in 2024; and triazoles like difenoconazole rose from 2.29% in 2021 to 9.26% in 2023 and 6.08% in 2024. Insecticides ranked second, representing 30% of top detections in 2021 and 2022 with combined frequencies of 6.87% and 6.59% respectively, rising to 40% in 2023 and 2024 at 21.15% and 10.24%; key insecticides included organophosphates such as chlorpyrifos at 2.32% in 2022 and 6.08% in 2023, and profenofos at 2.29% in 2021 and 3.59% in 2024, alongside neonicotinoids like clothianidin and imidacloprid, with the latter detected only in 2023 and 2024 at 4.89% and 1.95%. Finally, the sprouting inhibitor chlorpropham appeared sporadically, accounting for 10% of frequent detections in 2022 at 1.10% and in 2024 at 2.17%, signaling post-harvest treatments during onion storage and distribution. The observed shift in detection frequency, with a relative decrease in fungicides and an increase in insecticides, directly corresponds with evolving local agricultural practices and pest management strategies. For example, the declining detection of the fungicide carbendazim aligns with its increasing regulatory scrutiny and its gradual replacement by newer, more targeted alternatives in crop protection schedules. Conversely, the consistent detection of a compound like dimethomorph is linked to its specific and continued use against prevalent pathogens such as downy mildew in key regional crops.

The specific pesticides exceeding EU MRLs in our study are consistent with findings from earlier Egyptian studies, including profenofos and chlorpyrifos [24], as well as carbendazim, metalaxyl, chlorpyrifos, and profenofos reported by Malhat et al. [22,23]. Similar exceedances have been reported by Tadesse et al. [19] for chlorpyrifos and malathion in Ethiopian onions, Chau et al. [13] for cypermethrin (up to 43 times MRL) and difenoconazole in Vietnamese onions, Jardim and Caldas [20] for organophosphorus compounds in Brazilian onions, Osaili et al. [16] for profenofos and pyridaben in UAE onions, Ramadan et al. [17] for methomyl violations in Saudi Arabian onions, and Veiga-del-Baño et al. [15] for chlorpropham exceedances in Spanish onions.

Additionally, Chu et al. [9] identified fungicides as prevalent in Chinese onions, though with different compounds and 6% MRL exceedances occurring mainly from insecticides such as carbofuran and cyhalothrin. Horská et al. [14] also frequently detected azoxystrobin in Czech onions.

### 3.3. Incidence of Single and Multiple Residue Detections in Onion Samples

**Figure 2:**
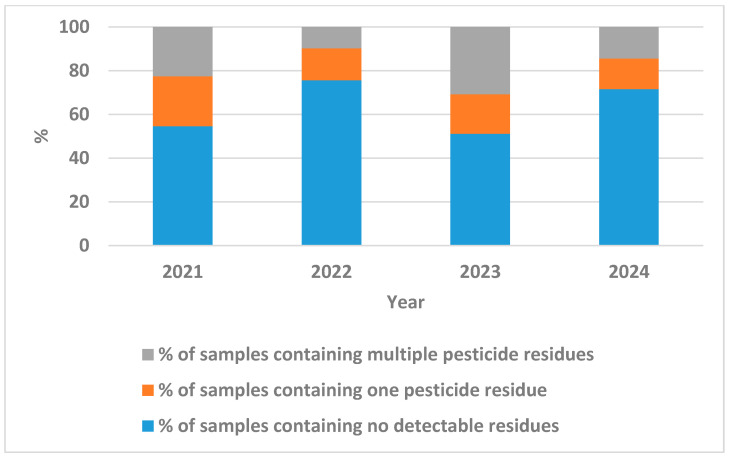
Distribution of single and multiple pesticide residues in Egyptian onions across four years.

The pesticide residue data reveals notable shifts in contamination patterns across the four-year period, particularly in the distribution between single and multiple residue occurrences, as shown in Figure 2. Single pesticide residues showed relative stability, fluctuating within a narrow range from 22.90% in 2021 to a low of 14.06% in 2024, with intermediate values of 14.65% (2022) and 17.99% (2023). In contrast, multiple pesticide residues exhibited more dramatic variations, starting at 22.52% in 2021, dropping sharply to 9.76% in 2022, representing a 56.6% decrease, before surging to a peak of 30.82% in 2023, the highest recorded level across all years. This 2023 spike in multiple residues coincided with the lowest proportion of residue-free samples (51.19%), suggesting a period of intensified pesticide application. The year 2024 showed improvement with multiple residues declining to 14.41%, approaching parity with single residue levels (14.06%), while residue-free samples recovered to 71.53%. The ratio of multiple to single residues varied considerably: approximately 1:1 in 2021, 0.67:1 in 2022, 1.71:1 in 2023, and 1.02:1 in 2024, indicating that 2023 was an outlier year where samples were more likely to contain multiple pesticides than single ones. This pattern suggests that contamination severity, rather than mere presence, fluctuated significantly, with 2022 and 2024 representing years of better pesticide management compared to 2021 and especially 2023.

While samples containing 9 pesticide residues were absent in 2021, they emerged at 0.04% in 2022, peaked at 0.40% in 2023, then declined to 0.09% in 2024, suggesting a tenfold increase from 2022 to 2023 followed by a 77.5% reduction (Table 2). Mid-range contamination (5–6 residues) showed progressive intensification, with 6-residue samples increasing from zero detection in 2021 to 0.67% by 2024, while 5-residue samples grew from 1.15% to peak at 3.84% in 2023 before moderating to 1.11% in 2024. The 3-residue category exhibited a notable decline from 6.49% in 2021 to 2.44% in 2022, then rebounded to 6.22% in 2023 before settling at 3.46% in 2024, while 4-residue samples peaked at 4.63% in 2023 from a baseline of 4.20% in 2021. The most substantial contamination category, samples with 2 pesticide residues, demonstrated relative stability around 10% (10.31% in 2021, 5.90% in 2022, 10.32% in 2023, and 6.52% in 2024), though the 2022 and 2024 values indicate approximately 40% reductions from peak years.

Nevertheless, the limited knowledge regarding interactions between multiple pesticides and human health makes their occurrence a cause for concern. Research [6,30,31] suggests that these exposures could lead to synergistic or additive toxic effects that heighten health risks. Climate change exacerbates pesticide contamination in agriculture by elevating pest pressure, which necessitates more frequent pesticide applications, and by modifying environmental conditions that slow down the breakdown of these chemicals. As a result, food crops end up accumulating greater levels of pesticide residues [32].

Comparative analyses with global studies reveal that our data’s temporal depth and emphasis on multi-residue complexity are relatively unique, with other regions showing varying but often less detailed instances of multiple residues. For instance, Chu et al. [9] reported multiple residues in 19.6% of Chinese onion samples, with up to 14 pesticides per sample. Similarly, Chau et al. [13] detected up to seven co-occurring residues in Vietnamese onions.

### 3.4. Health Risk Assessment

The chronic risk assessment of pesticide residues in onions, as evaluated using the EFSA PRIMo 3.1 model and presented in Table 3, reveals that exposure levels across diverse population groups remain well below the Acceptable Daily Intake (ADI) thresholds, with all calculated percentages (%ADI) substantially less than 100%, thereby indicating no appreciable long-term health risks from consumption of these contaminated onions. Notably, the highest %ADI values are observed for chlorpyrifos (ADI 0.001 mg/kg bw/day), ranging from 1.06% in the Portuguese general population to 2.27% in the GEMS/Food G06 cluster (encompassing populations from Armenia, Cuba, Egypt, Greece, Iran, Lebanon, and Turkey), which likely reflects higher per capita onion consumption patterns in these regions compared to others, such as the GEMS/Food G07 cluster (including Australia, Finland, France, and the United Kingdom (UK) where values are consistently lower. Other pesticides with relatively elevated %ADI include lambda-cyhalothrin (up to 0.727% in G06) and cypermethrin (up to 0.333% in G06), yet these figures underscore a broad margin of safety, as even the most conservative estimates, factoring in variations in dietary habits, body weights, and consumption data from clusters like GEMS/Food G10 and GEMS/Food G15, do not approach hazardous levels. Pesticides like clothianidin and azoxystrobin demonstrate negligible contributions (e.g., <0.004% across groups), highlighting the dominance of organophosphate and pyrethroid residues in overall exposure profiles. This comprehensive assessment across 45 distinct population groups strengthens the reliability of these findings and suggests that current agricultural practices and regulatory frameworks are effectively maintaining pesticide residues in onions at levels that ensure consumer safety across diverse dietary contexts and consumption patterns globally.

These results align with international studies confirming the safety of onion consumption despite pesticide residues. For instance, Ahn et al. [11] reported %ADI values up to 0.584% in South Korean onions and concluded that there were no significant long-term risks. Similarly, Bai et al. [8] found negligible chronic exposure to propiconazole in Chinese onions, with intakes well below ADI thresholds. Chu et al. [9] observed %ADI values below 100% in Chinese onions, while El-Sheikh et al. [24] reported acceptable levels with up to 21.13% ADI for profenofos. Additionally, Malhat et al. [22,23], Khaled et al. [33], and Gamal et al. [34] confirmed hazard indices and EDI values below ADI, indicating low risks. The European Food Safety Authority [21] assessed EU onions and found low chronic risks.

## 4. Conclusions

This four-year study of 5731 Egyptian onion samples from 2021 to 2024 highlights evolving pesticide residue dynamics, with detection of up to 67 pesticides in 2023, alongside variable compliance with EU MRLs. Residue-free samples peaked at 75.59% in 2022 but dropped to 51.19% in 2023, correlating with the highest MRL exceedance rate of 8.33% and multiple residue incidence of 30.82%. By 2024, improvements were evident, with 71.53% residue-free samples and exceedances reduced to 4.04%. The dramatic shift in pesticide class distribution—fungicides declining from 70% to 50% while insecticides increased from 30% to 40%—is likely driven by a complex combination of factors, including changing pest pressures, adaptive management strategies, regulatory requirements, and economic considerations influencing farmer choices. The elimination of carbendazim usage (from 20.99% to 2.35%) alongside the emergence of dimethomorph as a primary fungicide demonstrates active resistance management and regulatory compliance efforts. The sharp fluctuations in multiple residue occurrence, marked by a jump to 30.82% in 2023 and a drop to 14.41% in 2024, highlight the critical need for continuous monitoring programs to identify periods that may require intervention. Nevertheless, EFSA PRIMo 3.1-based chronic risk assessments across global population groups confirmed exposures far below ADI limits, with chlorpyrifos reaching a maximum 2.27% ADI in high-consumption clusters. These findings underscore the need for sustained regulatory enhancements to minimize exceedances while affirming overall consumer safety.

## Figures and Tables

**Table 1:** Yearly comparison of predominant pesticides detected in Egyptian onions (2021–2024).

2021 (*n* = 262)					2022 (*n* = 2458)				
Pesticide	Frequency%	Mean (mg/kg)	Range (Min.–Max.)	EU MRL (mg/kg)	Pesticide	Frequency%	Mean (mg/kg)	Range (Min.–Max.)	EU MRL (mg/kg)
Carbendazim	20.99	0.006	<LOQ–0.025	0.10	Carbendazim	10.09	0.011	<LOQ–0.197	0.10
Azoxystrobin	20.99	0.005	<LOQ–0.013	10.00	Metalaxyl	5.57	0.005	<LOQ–0.025	0.03
Metalaxyl	10.69	0.005	<LOQ–0.027	0.03	Azoxystrobin	3.86	0.006	<LOQ–0.030	10.00
Boscalid	4.96	0.005	<LOQ–0.009	5.00	Cypermethrin	2.97	0.007	<LOQ–0.057	0.10
Dimethomorph	2.67	0.005	<LOQ–0.006	0.60	Chlorpyrifos	2.32	0.008	<LOQ–0.064	0.01
Difenoconazole	2.29	0.005	<LOQ–0.005	0.50	Thiophanate-methyl	2.24	0.020	<LOQ–0.231	0.10
Lambda-Cyhalothrin	2.29	0.005	<LOQ–0.006	0.20	Difenoconazole	1.79	0.007	<LOQ–0.049	0.50
Imazalil	2.29	0.029	<LOQ–0.054	0.01	Dimethomorph	1.51	0.008	<LOQ–0.042	0.60
Profenofos	2.29	0.005	<LOQ–0.005	0.02	Clothianidin	1.30	0.005	<LOQ–0.026	0.01
Clothianidin	2.29	0.005	<LOQ–0.005	0.01	Chlorpropham	1.10	0.014	<LOQ–0.049	0.01
**2023 (*n* = 756)**					**2024 (*n* = 2255)**				
**Pesticide**	**Frequency%**	**Mean (mg/kg)**	**Range (Min.** **–** **Max.)**	**EU MRL (mg/kg)**	**Pesticide**	**Frequency%**	**Mean (mg/kg)**	**Range (Min.** **–** **Max.)**	**EU MRL (mg/kg)**
Azoxystrobin	18.78	0.018	<LOQ–0.213	10.00	Azoxystrobin	11.97	0.030	<LOQ–2.204	10.00
Metalaxyl	17.33	0.019	<LOQ–0.277	0.03	Metalaxyl	8.07	0.011	<LOQ–0.284	0.03
Dimethomorph	16.67	0.037	<LOQ–0.297	0.60	Dimethomorph	7.23	0.046	<LOQ–1.102	0.60
Difenoconazole	9.26	0.011	<LOQ–0.079	0.50	Difenoconazole	6.08	0.022	<LOQ–0.559	0.50
Chlorpyrifos	6.08	0.030	<LOQ–0.297	0.01	Profenofos	3.59	0.032	<LOQ–0.336	0.02
Carbendazim	5.56	0.020	<LOQ–0.281	0.10	Lambda-Cyhalothrin	2.75	0.015	<LOQ–0.225	0.20
Boscalid	5.42	0.020	<LOQ–0.102	5.00	Carbendazim	2.35	0.011	<LOQ–0.070	0.10
Cypermethrin	5.29	0.022	<LOQ–0.183	0.10	Chlorpropham	2.17	0.021	<LOQ–0.125	0.01
Lambda-Cyhalothrin	4.89	0.024	<LOQ–0.205	0.20	Cypermethrin	1.95	0.010	<LOQ–0.064	0.10
Imidacloprid	4.89	0.015	<LOQ–0.089	0.01	Imidacloprid	1.95	0.040	<LOQ–0.304	0.01

**Table 2:** Frequency of multiple pesticide residues in Egyptian market onions: Distribution by number of residues per sample (2021–2024).

Year	2021	2022	2023	2024
Multiple Pesticides	%	%	%	%
9 pesticide residues	0.00	0.04	0.40	0.09
8 pesticide residues	0.00	0.04	0.40	0.13
7 pesticide residues	0.38	0.00	1.06	0.40
6 pesticide residues	0.00	0.08	2.91	0.67
5 pesticide residues	1.15	0.33	3.84	1.11
4 pesticide residues	4.20	0.98	4.63	1.86
3 pesticide residues	6.49	2.44	6.22	3.46
2 pesticide residues	10.3	5.90	10.32	6.52
1 pesticide residue	22.9	14.7	17.9	14.1

**Table 3:** Chronic dietary exposure to pesticides in Egyptian onions: International Estimated Daily Intake (IEDI) and % Acceptable Daily Intake (%ADI) across diverse populations.

Pesticide (ADI)	MS Diet	GEMS/Food G06	Romania General	GEMS/Food G10	GEMS/Food G15	GEMS/Food G08	Sweden General	Finland 3-Year-Old Children	GEMS/Food G07	Portugal General
Azoxystrobin (0.2)	IEDI	2.27 × 10^−2^	2.25 × 10^−2^	1.75 × 10^−2^	1.56 × 10^−2^	1.53 × 10^−2^	1.35 × 10^−2^	1.17 × 10^−2^	1.06 × 10^−2^	1.06 × 10^−2^
	% of ADI	1.14 × 10^−2^	1.13 × 10^−2^	8.77 × 10^−3^	7.78 × 10^−3^	7.64 × 10^−3^	6.75 × 10^−3^	5.86 × 10^−3^	5.31 × 10^−3^	5.28 × 10^−3^
Boscalid (0.04)	IEDI	1.51 × 10^−2^	1.50 × 10^−2^	1.17 × 10^−2^	1.04 × 10^−2^	1.02 × 10^−2^	9.00 × 10^−3^	7.82 × 10^−3^	7.08 × 10^−3^	7.03 × 10^−3^
	% of ADI	3.78 × 10^−2^	3.75 × 10^−2^	2.92 × 10^−2^	2.59 × 10^−2^	2.55 × 10^−2^	2.25 × 10^−2^	1.95 × 10^−2^	1.77 × 10^−2^	1.76 × 10^−2^
Carbendazim (0.02)	IEDI	1.51 × 10^−2^	1.50 × 10^−2^	1.17 × 10^−2^	1.04 × 10^−2^	1.02 × 10^−2^	9.00 × 10^−3^	7.82 × 10^−3^	7.08 × 10^−3^	7.03 × 10^−3^
	% of ADI	7.57 × 10^−2^	7.50 × 10^−2^	5.85 × 10^−2^	5.18 × 10^−2^	5.10 × 10^−2^	4.50 × 10^−2^	3.91 × 10^−2^	3.54 × 10^−2^	3.52 × 10^−2^
Chlorpropham (0.05)	IEDI	1.59 × 10^−2^	1.58 × 10^−2^	1.23 × 10^−2^	1.09 × 10^−2^	1.07 × 10^−2^	9.45 × 10^−3^	8.21 × 10^−3^	7.43 × 10^−3^	7.39 × 10^−3^
	% of ADI	3.18 × 10^−2^	3.15 × 10^−2^	2.46 × 10^−2^	2.18 × 10^−2^	2.14 × 10^−2^	1.89 × 10^−2^	1.64 × 10^−2^	1.49 × 10^−2^	1.48 × 10^−2^
Chlorpyrifos (0.001)	IEDI	2.27 × 10^−2^	2.25 × 10^−2^	1.75 × 10^−2^	1.56 × 10^−2^	1.53 × 10^−2^	1.35 × 10^−2^	1.17 × 10^−2^	1.06 × 10^−2^	1.06 × 10^−2^
	% of ADI	2.27 × 10^0^	2.25× 10^0^	1.75× 10^0^	1.56× 10^0^	1.53× 10^0^	1.35× 10^0^	1.17× 10^0^	1.06× 10^0^	1.06× 10^0^
Clothianidin (0.097)	IEDI	3.78 × 10^−3^	3.75 × 10^−3^	2.92 × 10^−3^	2.59 × 10^−3^	2.55 × 10^−3^	2.25 × 10^−3^	1.95 × 10^−3^	1.77 × 10^−3^	1.76 × 10^−3^
	% of ADI	3.90 × 10^−3^	3.87 × 10^−3^	3.01 × 10^−3^	2.67 × 10^−3^	2.63 × 10^−3^	2.32 × 10^−3^	2.01 × 10^−3^	1.82 × 10^−3^	1.81 × 10^−3^
Cypermethrin (0.005)	IEDI	1.67 × 10^−2^	1.65 × 10^−2^	1.29 × 10^−2^	1.14 × 10^−2^	1.12 × 10^−2^	9.90 × 10^−3^	8.60 × 10^−3^	7.79 × 10^−3^	7.74 × 10^−3^
	% of ADI	3.33 × 10^−1^	3.30 × 10^−1^	2.57 × 10^−1^	2.28 × 10^−1^	2.24 × 10^−1^	1.98 × 10^−1^	1.72 × 10^−1^	1.56 × 10^−1^	1.55 × 10^−1^
Difenoconazole (0.01)	IEDI	1.67 × 10^−2^	1.65 × 10^−2^	1.29 × 10^−2^	1.14 × 10^−2^	1.12 × 10^−2^	9.90 × 10^−3^	8.60 × 10^−3^	7.79 × 10^−3^	7.74 × 10^−3^
	% of ADI	1.67 × 10^−1^	1.65 × 10^−1^	1.29 × 10^−1^	1.14 × 10^−1^	1.12 × 10^−1^	9.90 × 10^−2^	8.60 × 10^−2^	7.79 × 10^−2^	7.74 × 10^−2^
Dimethomorph (0.05)	IEDI	3.48 × 10^−2^	3.45 × 10^−2^	2.69 × 10^−2^	2.38 × 10^−2^	2.34 × 10^−2^	2.07 × 10^−2^	1.80 × 10^−2^	1.63 × 10^−2^	1.62 × 10^−2^
	% of ADI	6.96 × 10^−2^	6.90 × 10^−2^	5.38 × 10^−2^	4.77 × 10^−2^	4.69 × 10^−2^	4.14 × 10^−2^	3.60 × 10^−2^	3.26 × 10^−2^	3.24 × 10^−2^
Imazalil (0.025)	IEDI	2.19 × 10^−2^	2.18 × 10^−2^	1.70 × 10^−2^	1.50 × 10^−2^	1.48 × 10^−2^	1.31 × 10^−2^	1.13 × 10^−2^	1.03 × 10^−2^	1.02 × 10^−2^
	% of ADI	8.78 × 10^−2^	8.70 × 10^−2^	6.78 × 10^−2^	6.01 × 10^−2^	5.91 × 10^−2^	5.22 × 10^−2^	4.53 × 10^−2^	4.11 × 10^−2^	4.08 × 10^−2^
Imidacloprid (0.06)	IEDI	3.03 × 10^−2^	3.00 × 10^−2^	2.34 × 10^−2^	2.07 × 10^−2^	2.04 × 10^−2^	1.80 × 10^−2^	1.56 × 10^−2^	1.42 × 10^−2^	1.41 × 10^−2^
	% of ADI	5.05 × 10^−2^	5.00 × 10^−2^	3.90 × 10^−2^	3.46 × 10^−2^	3.40 × 10^−2^	3.00 × 10^−2^	2.61 × 10^−2^	2.36 × 10^−2^	2.34 × 10^−2^
Lambda-Cyhalothrin (0.0025)	IEDI	1.82 × 10^−2^	1.80 × 10^−2^	1.40 × 10^−2^	1.24 × 10^−2^	1.22 × 10^−2^	1.08 × 10^−2^	9.38 × 10^−3^	8.50 × 10^−3^	8.44 × 10^−3^
	% of ADI	7.27 × 10^−1^	7.20 × 10^−1^	5.61 × 10^−1^	4.98 × 10^−1^	4.89 × 10^−1^	4.32 × 10^−1^	3.75 × 10^−1^	3.40 × 10^−1^	3.38 × 10^−1^
Metalaxyl (0.08)	IEDI	1.44 × 10^−2^	1.43 × 10^−2^	1.11 × 10^−2^	9.85 × 10^−3^	9.68 × 10^−3^	8.55 × 10^−3^	7.43 × 10^−3^	6.73 × 10^−3^	6.68 × 10^−3^
	% of ADI	1.80 × 10^−2^	1.78 × 10^−2^	1.39 × 10^−2^	1.23 × 10^−2^	1.21 × 10^−2^	1.07 × 10^−2^	9.28 × 10^−3^	8.41 × 10^−3^	8.35 × 10^−3^
Profenofos (0.03)	IEDI	2.42 × 10^−2^	2.40 × 10^−2^	1.87 × 10^−2^	1.66 × 10^−2^	1.63 × 10^−2^	1.44 × 10^−2^	1.25 × 10^−2^	1.13 × 10^−2^	1.13 × 10^−2^
	% of ADI	8.07 × 10^−2^	8.00 × 10^−2^	6.24 × 10^−2^	5.53 × 10^−2^	5.43 × 10^−2^	4.80 × 10^−2^	4.17 × 10^−2^	3.78 × 10^−2^	3.75 × 10^−2^
Thiophanate-methyl (0.02)	IEDI	1.51 × 10^−2^	1.50 × 10^−2^	1.17 × 10^−2^	1.04 × 10^−2^	1.02 × 10^−2^	9.00 × 10^−3^	7.82 × 10^−3^	7.08 × 10^−3^	7.03 × 10^−3^
	% of ADI	7.57 × 10^−2^	7.50 × 10^−2^	5.85 × 10^−2^	5.18 × 10^−2^	5.10 × 10^−2^	4.50 × 10^−2^	3.91 × 10^−2^	3.54 × 10^−2^	3.52 × 10^−2^

GEMS/Food G06: General population (Armenia, Cuba, Egypt, Greece, Iran, Lebanon, and Turkey). GEMS/Food G07: General population (Australia, Bermuda, Finland, France, Iceland, Luxembourg, Norway, Switzerland, the UK, and Uruguay). GEMS/Food G08: General population (Austria, Germany, Poland, and Spain). GEMS/Food G10: General population (Belarus, Bulgaria, Canada, Croatia, Cyprus, Estonia, Italy, Japan, Latvia, Malta, New Zealand, the Republic of Korea, the Russian Federation, and the USA). GEMS/Food G15: General population (the Czech Republic, Denmark, Hungary, Ireland, Lithuania, Portugal, Romania, Serbia, Slovakia, Slovenia, and Sweden).

## Data Availability

The original contributions presented in the study are included in the article/Supplementary Material. Further inquiries can be directed to the corresponding author.

## Supplementary Materials

The following supporting information can be downloaded at https://www.mdpi.com/article/10.3390/jox15060192/s1. Table S1: LC-MS/MS optimization parameters including molecular masses to charge ratio (*m*/*z*) in the quads (q1&q3) retention time (RT), decluster potential (DP), entrance potential (EP), collision energy (CE) and collision cell exit potential (CXP) for all target MRM transitions of LC-Compounds.; Table S2: GC-MS/MS optimization parameters including retention time (RT), molecular masses to charge ratio (*m*/*z*) in the quads (q1&q3), and collision energy (CE) for all target MRM transitions of GC-Compounds.; Table S3: Limits of quantification (LOQ), linearity (R2), average recoveries (Rec.), and relative standard deviations (RSD) for 430 pesticides analyzed in onions.; Figure S1: LC-MS/MS chromatogram of the analytical standard of LC analytes at 0.1 mg/kg.; Figure S2: GC-MS/MS chromatogram of analytical standard of GC analytes at 0.1 mg/kg.
