# Supplementary material for: Temporal Trends, Multiple Residue Incidence, and Chronic Health Risk of Pesticides in Egyptian Onions: A Four-Year Market Surveillance
Source: J Xenobiot. 2025 Nov 10;15(6):192. doi: 10.3390/jox15060192 (PMC12641999; doi:10.3390/jox15060192)
Supplement: Supplementary file 1 [file jox-15-00192-s001.zip › jox-3937740-supplementary.pdf]

Table S1: LC-MS/MS optimization parameters including molecular masses to charge ratio (m/z) in the quads (q1&q3) retention time (RT), decluster potential (DP), Entrance Potential (EP), collision energy (CE) and Collision Cell Exit Potential (CXP) for all target MRM transitions of LC-Compounds.

Pesticide Name	Chemical class and type	Q1 (da)	Q3 (da)	RT (min)	DP (volts)	EP (volts)	CE (volts)	CXP (volts)
3-Hydroxycarbofuran_1*	Metabolite	238.1	181.1	5.49	59	12	15	6.5
3-Hydroxycarbofuran_2**		238.1	163.1	5.5	82	10	21	4
Abamectin_1	Avermectin insecticide/ acaricide	890.5	567.4	15.5	81	10	19	8
Abamectin_2		890.5	305.3	15.5	81	10	37	16
Acephate_1	Organophosphorus insecticide	184.1	124.9	1.12	55	10	25	6.5
Acephate_2		184.1	94.9	1.12	61	8	31	4
Acequinocyl_1	Quinone acaricide/ insecticide	402.2	343.1	17.1	71	8.5	19	20
Acequinocyl_2		402.2	189	17.1	71	10	39	10
Acetamiprid_1	Neonicotinoid insecticide	223.2	126.1	5.61	76	3.5	29	4
Acetamiprid_2		223.2	99.1	5.59	76	3.5	47	4
Afidopyropen_1	Pyropene insecticide	594	202	11.5	90	10	45	2
Afidopyropen_2		594	148	11.5	90	10	59	4
Aldicarb_1	Carbamate insecticide	208.1	116	6.6	51	2	11	3
Aldicarb_2		208.1	88.9	6.59	51	2	20	3
Aldicarb-sulfone_1	Metabolite	223.1	148	2.19	70	10	12	3
Aldicarb-sulfone_2		223.1	86.1	2.21	70	10	20	3
Aldicarb-sulfoxide_1	Metabolite	207.1	132.1	1.57	56	9	11	4
Aldicarb-sulfoxide_2		207.1	89.1	1.67	56	9	17	4
Amidosulfuron_1	Sulfonylurea herbicide	370	261	8.99	61	10	19	10
Amidosulfuron_2		370	217.9	8.98	61	9.5	31	6.5
Aminocarb_1	Carbamate insecticide	209.1	152.1	0.89	56	8.5	19	6.5
Aminocarb_2		209.2	137.1	1.23	70	10	32	3
Amitrole_1	Triazole herbicide	85	58.2	0.54	86	11.5	29	6.5
Amitrole_2		85	43.4	0.45	86	11.5	33	6.5
Azamethiphos_1	Organophosphorus Insecticide	325	183	7.8	59	10	21	6.5
Azamethiphos_2		325	139	7.8	56	10	33	6
Azimsulfuron_1	Sulfonylurea herbicide	425.1	182.1	9.95	71	8.5	23	6.5
Azoxystrobin_1	Strobilurin fungicide	404.1	372.1	10.9	71	5	19	6
Azoxystrobin_2		404.1	344.1	10.9	71	5	27	4
Barban_1	Carbamate ester herbicide	258	178	10.5	105.33	10	15	12
Barban_2		258	143	10.5	105.33	10	29	12
Beflubutamid_1	Amide herbicide	356.1	91.1	10.8	51	10	53	14
Beflubutamid_2		356	162	10.8	71	10	39	24
Bendiocarb_1	Carbamate insecticide	224	167.2	8.04	61	9.5	13	4
Bendiocarb_2		224	109.1	8.03	61	9.5	23	4

Bensulfuron-methyl_1	Sulfonylurea herbicide	411.1	148.9	10.4	86	10	27	6.5
Bensulfuron-methyl_2		411	119	10.5	81	10	51	6
Bentazon_1	Benzothiazinone herbicide	241	199	8.05	96	10	19	6
Bentazon_2		241	107	8.06	96	10	39	4
Benthiavalicarb isopropyl_1	Carbamate fungicide	382.1	180.1	11.5	112	10	53	24
Benthiavalicarb isopropyl_2		382.1	116.1	11.5	77	10	39	18
Bispyribac_1	Pyrimidinyloxybenzoic herbicide	431	275	11	82	10	17	6.5
Bispyribac_2		431	413	11	82	10	25	6.5
Benzoximate_1	Bridged diphenyl acaricide	364	199.1	13	56	4.5	17	4
Benzoximate_2		364	105.1	13	56	4.5	31	4
Boscalid_1	Carboxamide fungicide	343	307	11	94	10	27	6.5
Boscalid_2		343	140	11	116	10	27	6
Bromacil_1	Uracil herbicide	261	205	7.78	61	10	19	6.5
Bromacil_2		261	188	7.78	61	10	37	7
Butocarboxim_1	Carbamate insecticide	213	156	6.49	100	10	10	8
Butocarboxim_2		213	75	6.47	100	10	10	8
Carbaryl_1	Carbamate insecticide	202.1	144.9	8.46	51	10	15	6.5
Carbaryl_2		202.1	127.1	8.46	71	10	39	3
Carbendazim_1	Benzimidazole fungicide	192.2	132.1	2.45	76	4.5	41	4
Carbendazim_2		192.2	160.2	2.46	76	4.5	27	4
Carbetamide_1	Carbanilate herbicide	237	192	7.33	66	10	13	7
Carbetamide_2		237.1	118.1	7.33	64	7	19	6.5
Carbofuran_1	Carbamate insecticide	222.101	165.2	8.05	56	9.5	17	6.5
Carbofuran_2		222.101	123	8.05	70	10	29	2
Carbosulfan_1	Carbamate insecticide	381.2	160.2	15.6	72	10	20	3
Carbosulfan_2		381.2	118.1	15.6	76	8.5	25	6.5
Carboxin_1	Oxathiin fungicide	236.1	142.9	8.45	64	8	21	6.5
Carboxin_2		236	87	8.46	61	10	33	4
Chlorantranilinprole_1	Anthranilic diamide insecticide	484	453	10.3	61	6	23	20
Chlorantranilinprole_2		484	286	10.3	61	6	21	12
Chlorbromuron_1	Phenylurea herbicide	293	204	10.7	81	10	21	7
Chlorbromuron_2		292.9	182.1	10.7	86	11.5	23	6.5
Chlorfluazuron_1	Benzoylurea insecticide	540	383	15	121	10	27	12
Chlorfluazuron_2		539.9	158	15	96	9.5	27	6.5
Chloridazon_1	Pyridazinone herbicide	222	104	5.33	101	10	31	6
Chloridazon_2		222	92.2	5.32	79	10	35	6.5
Chloroxuron_1	Phenylurea herbicide	291	218	11.3	86	10	33	7
Chloroxuron_2		291.1	72	11.3	89	10	41	6.5
Chlorsulfuron_1		358	167	9.02	91	10	25	7

Chlorsulfuron_2	Sulfonylurea herbicide	358	141	9.01	71	10	23	6.5
Chlortoluron_1	Phenylurea herbicide	213.1	72.2	8.9	81	6	31	4
Chlortoluron_2		213.1	46.2	8.9	81	6	27	4
Chromafenozide_1	Diacylhydrazine insecticide	395	339	12	32	5	10	13
Chromafenozide_2		395	175	12	32	5	20	13
Cinidon-ethyl_1	Dicarboximide herbicide	411.1	348	13.9	81	9	29	6.5
Cinidon-ethyl_2		394	107	15.8	81	10	55	12
Cinosulfuron_1	Sulfonylurea herbicide	414.1	215	8.15	71	10	21	7
Cinosulfuron_2		414.1	182.9	8.15	71	10	23	6.5
Clethodim_1	Cyclohexanedione herbicide	360.1	268.2	13.5	84	8.5	17	6.5
Clethodim_2		360	164	13.5	81	10	25	6
Clofentezine_1	Tetrazine insecticide	303	138	12.8	96	10	21	6
Clofentezine_2		303.1	102.1	12.8	76	8	47	6.5
Clopyralid_1	Pyridine herbicide	191.9	146	1.75	54	10.5	29	8
Clopyralid_2		191.9	110.1	1.82	54	12	47	6
Clothianidin_1	Neonicotinoid insecticide	250	169.1	4.76	94	10	19	6.5
Clothianidin_2		250	132	4.72	70	10	30	4
Coumaphos_1	Organophosphorus insecticide	363	307	12.8	96	9	23	6.5
Coumaphos_2		363	227	12.8	84	12	35	6.5
Coumatetralyl_1	Coumarin rodenticide	293.1	175	11.7	106	11	29	6.5
Coumatetralyl_2		293.2	91.1	11.7	66	10	55	4
Cyanophos_1	Organophosphorus insecticide	244	134	10.7	85	10	33	4
Cyanophos_2		261	125	10.4	58	10	27	6.5
Cyantraniliprole_1	Diamide insecticide	475.1	444	9.11	70	6	27	29
Cyantraniliprole_2		475.1	286	9.11	70	6	18	20
Cyazofamid_1	Imidazole fungicide	325	261	11.9	76	10	15	10
Cyazofamid_2		325	108	12	76	9.5	19	6.5
Cycloxydim_1	Cyclohexanedione herbicide	326	280	13.4	111	10	21	32
Cycloxydim_2		326	180	13.4	111	10	29	26
Cyflumetofen_1	Bridged diphenyl insecticide	465	249.2	13.9	30	8	18.5	7.3
Cyflumetofen_2		465	173	13.9	30	8	27.7	5
Cyhalofop-butyl_1	Aryloxyphenoxypropionate herbicide	375.1	256.1	13.5	76	11.5	21	6.5
Cyhalofop-butyl_2		375.1	120	13.5	51	11.5	43	6.5
Cymoxanil_1	Cyanoacetamide oxime fungicide	199.1	128.1	5.79	61	7.5	13	4
Cymoxanil_2		199.1	111.1	5.79	61	7.5	23	4
Cyphenothrin_1	Pyrethroid insecticide	393.2	151.2	16.8	21	9.5	21	8
Cyphenothrin_2		393.2	123.1	16.8	21	12	33	6
Cyromazine_1	Triazine insecticide/ acaricide	167.1	125	0.58	84	8.5	25	6.5
Cyromazine_2		167	108	0.57	81	10	29	6
Deltamethrin_1	Pyrethroid insecticide	523	281	15.1	56	10	23	10
Deltamethrin_2		523	181	15.1	56	10	51	7

Desmedipham_1	Carbamate herbicide	318.1	182.2	10.2	64	10	19	6.5
Desmedipham_2		318	136	10.4	56	10	33	6
Desmethyl-pirimicarb_1	Metabolite	225	168	8.02	56	10	19	7
Desmethyl-pirimicarb_2		225.1	72	4.14	56	8.5	27	6.5
Dichlofluanid_1	Sulphamide fungicide	350	123	11.8	32	6.5	37	15
Dichlofluanid_2		350	224	11.8	31	6.5	20	15
Dichlorvos_1	Organophosph orus insecticide	220.9	127.2	7.68	76	11	23	4
Dichlorvos_2		220.9	109.1	7.66	76	11	25	4
Diclofop-methyl_1	Aryloxyphenoxypropionate herbicide	358	281	13.7	66	10	21	6.5
Diclofop-methyl_2		358	120	13.7	72	10	41	6
Dicrotophos_1	Organophosph orus insecticide	238.1	127.1	4.75	56	9.5	23	6.5
Dicrotophos_2		238	112	4.73	66	10	17	6.5
Diafenthiuron_1	Thiourea insecticide	385	278	14.7	112	10	45	10
Diafenthiuron_2		385	329.2	14.7	112	10	45	10
Diflubenzuron_1	Benzoylurea herbicde	311	158.2	12	66	8	19	4
Diflubenzuron_2		311	141.2	12	66	8	47	4
Dimethoate_1	Organophosph ate insecticide	230	199.1	5.13	61	7.5	13	4
Dimethoate_2		230	125	5.13	61	7.5	29	4
Dinotefuran 1	Neonicotinoid insecticide	203.1	129.1	1.57	58	10	20	4
Dinotefuran 2		203.1	114.1	1.61	58	10	33	4
Diphacinone_1	Indandione rodenticide	341.2	263.1	10.2	56	10	19	18
Diphacinone_2		341.2	235.1	10.2	56	10	27	14
Disulfoton_1	Organophosph ate insecticide	275.1	89	14.9	61	6.5	15	8
Disulfoton_2		275.1	61	13	61	6.5	49	4
Diuron_1	Phenylurea herbicide	233.01	160.2	8.91	76	11	33	4
Diuron_2		233.01	72	8.92	76	11	33	4
Dodine_1	Guanidine fungicide	228.3	60.1	11.9	91	11	37	6.5
Dodine_2		228.3	57	10.6	26	10	43	8
Emamectin_1	Bio-insecticide	886.5	158.1	13.5	91	10	53	8
Emamectin_2		886.5	82	13.5	91	10	125	12
EPN_1	Organophosph ate insecticide	324	296	13.3	76	8	19	6
EPN_2		324	156.9	13.3	86	10	29	6.5
Eprinomectin_1	Avermectin antiparasitic	914.5	468.2	15.2	106	10	17	8
Eprinomectin_2i		914.5	186.1	15.2	76	10	19	8
Ethiofencarb_1	Carbamate insecticide	226.1	164.1	8.75	61	10	11	3
Ethiofencarb_2		226.1	107.2	8.74	54	9.5	21	6.5
Ethiofencarb-sulfone_1	Metabolite	258.1	200.9	4.56	46	10	11	10
Ethiofencarb-sulfone_2		258.1	107.2	4.55	46	10	27	16
Ethiofencarb-sulfoxide_1	Metabolite	242.1	107.1	4.72	61	10	23	4
Ethiofencarb-sulfoxide_2		242.1	185	4.72	61	10.5	13	10
Ethiprole_1	Phenylpyrazol e insecticide	396.9	350.9	13.9	111	10	29	8

Ethirimol_1	Pyrimidine fungicide	210	140	5.63	91	10	31	6
Ethirimol_2		210.2	98.1	5.64	86	12	37	6.5
Ethofumesate_1	Benzofuran herbicide	287.1	259.1	10.8	76	10	15	4
Ethofumesate_2		287.1	121.1	10.8	76	10	21	4
Ethoprophos_1	Organophosphate insecticide/ nematicide	243	131	11.7	66	10	29	6.5
Ethoprophos_2		243	97	11.7	71	10	41	4
Ethylenethiourea (ETU)_1	Metabolite	103	60	0.57	69	10	47	6.5
Etoxazole_1	Diphenyl oxazoline insecticide	360.2	141	14.6	76	9	37	6.5
Etoxazole_2		360.1	57.2	14.6	75	9	45	6.5
Famoxadone_1	Oxazole fungicide	392	331	12.7	51	10	15	12
Famoxadone_2		392.2	238	12.7	54	8.5	23	6.5
Fenazaquin_1	Quinazoline acaricide	307.2	161.2	15	76	10.5	31	6.5
Fenazaquin_2		307	147	15	61	10	25	6
Fenbutatin oxide_1	Organotin insecticide	519	351	17	129.7	10	45.2	4
Fenbutatin oxide_2		519	197	17	129.7	10	63.9	4
Fenhexamid_1	Analide fungicide	302.1	97.2	11.5	101	12	33	6.5
Fenhexamid_2		302	55	11.5	131	10	57	4
Fenoxaprop-P-ethyl_1	Aryloxyphenoxypropionate herbicide	362.1	288.1	13.8	71	10.5	23	6.5
Fenoxaprop-P-ethyl_2		362	121	13.5	71	10	37	6
Fenoxycarb_1	Carbamate insecticide	302.2	116.2	12.1	68	10	16	3
Fenoxycarb_2		302.1	88	12.1	84	10	29	6.5
Fenpropidin_1	Piperidine fungicide	274.2	147.1	9.32	71	10	37	6.5
Fenpropidin_2		274	117	9.32	91	10	65	6
Fenpyrazamine 1	Pyrazolium fungicide	332.1	230	11.6	32	6	25	12
Fenpyrazamine 2		332.1	216.1	11.6	32	6	36.5	11
Fenpyroximate_1	Pyrazolium insecticide/ acaricide	422.1	366.2	14.8	76	6	23	6
Fenpyroximate_2		422.1	107.1	14.8	76	6	75	4
Fipronil_1	Phenylpyrazole insecticide/ acaricide	453.9	368.1	12.3	54	7	29	6.5
Fipronil_2		439	369.8	12.3	100	10	25	36
Flonicamid_1	Pyridine insecticide	230	97.8	3.13	76	12	47	4
Flonicamid_2		230	203.2	3.11	76	12	25	4
Florasulam_1	Triazolopyrimidine herbicide	360	192	7.14	121	10	23	7
Florasulam_2		360	129.1	7.13	96	11	29	6.5
Flubendiamide 1	Phthalamide insecticide	683.1	408	12.7	100	10	15	4
Flubendiamide 2		683.1	274.1	12.7	100	10	20	4
Fludioxonil_1	Phenylpyrrole fungicide	266	229	11	65	10	20	4
Fludioxonil_2		266	158	11	65	10	51	12
Flufenacet_1	Anilide herbicide	364.1	194.2	11.8	61	7	17	4
Flufenacet_2		364.1	152.2	11.9	61	7	25	4
Flufenoxuron_1		489.1	158.2	14.7	81	7	25	4

Flufenoxuron_2	Benzoylurea insecticide/ acaricide	489.1	141.2	14.7	81	7	65	4
Flumetsulam_1	Sulfonanilide herbicide	326.1	129.2	5.5	81	11.5	33	6.5
Flumetsulam_2		326.1	109	5.49	81	10	90	4
Fluometuron_1	Phenylurea herbicide	233	145.2	8.9	76	11	45	4
Fluometuron_2		233	72	8.9	76	11	37	4
Fluopicolide_1	Benzamide fungicide	383	173	11.3	77	10	31	6
Fluopicolide_2		383	145	11.3	77	10	73	6
Flupyradifurone_1	Organofluoride insecticide	289	245	5.66	149	7.8	21	12
Flupyradifurone_2		289	126	5.65	149	7.8	27	15
Fluopyram 1	Benzamide fungicide	397	208	11.7	40	10	35	7
Fluopyram 2		397	173	11.7	40	10	42	7
Fluquinconazole_1	Triazole fungicide	376	349	11.5	91	10	25	14
Fluquinconazole_2		376	307.1	11.5	94	9	33	6.5
Fluroxypyr_1	Pyridine herbicide	255.1	209.1	14.5	66	7	19	4
Fluroxypyr_2		255.1	181	14.5	66	7	31	4
Fluroxypyr-meptyl_1	Pyridine herbicide	367.1	255	14.5	71	9.5	17	6.5
Fluroxypyr-meptyl_2		367	209	14.5	71	10	29	7
Flurtamone_1	Furan herbicide	334.1	247.1	10.9	84	11	35	6.5
Flurtamone_2		334	178	10.9	76	10	57	7
Flusilazole_1	Triazole fungicide	316	247.1	12.1	76	10	23	4
Flusilazole_2		316	165.1	12.1	76	10	39	4
Flutolanil_1	Benzanilide fungicide	324	262.2	11.3	76	10	23	4
Flutolanil_2		324	242.2	11.3	76	10	31	4
Flutriafol_1	Triazole fungicide	302.1	122.9	9.52	84	8	39	6.5
Flutriafol_2		302	109	9.52	86	10	43	6
Fluxapyroxad_1	Pyrazole fungicide	382	342.1	11	110	10	53	24
Fluxapyroxad_2		382	314.1	11	110	10	39	18
Folpet_1	Phthalimide fungicide	315	163	11.5	41	10	19	7
Folpet_2		315	130	10.7	41	10	39	6
Fomesafen_1	Diphenyl ether herbicide	456	344.1	11.3	89	10	21	6.5
Foramsulfuron_1	Sulfonylurea herbicide	453	182.2	9.02	71	10	35	12
Foramsulfuron_2		453	272	9.02	76	10	21	12
Formetanate_1	Formamidine insecticide/ acaricide	222.1	165.1	9.07	61	12	19	6.5
Formetanate_2		222	120	1.04	76	5	37	6.5
Fosthiazate_1	Organophosphate insecticide/ nematicide	284	104	9.07	101	10	27	6
Fosthiazate_2		284	228	9.08	101	10	15	8
Halauxifen_1	Pyridine herbicide	345	285	10.8	65	6.8	29	15
Halauxifen_2		345	235	10.8	65	6.8	57	29
Halosulfuron-methyl_1	Sulfonylurea herbicide	435	182.1	11.7	76	10	27	6.5

Hexaflumuron_1	Phenylurea herbicide	461.1	158.2	13.7	76	8.5	25	4
Hexaflumuron_2		461.1	141.1	13.7	76	8.5	65	4
Hexazinone_1	1,2,4-triazinone herbicide	253.2	171.1	7.94	56	10.5	21	6.5
Hexazinone_2		253.2	71.1	7.94	86	4.5	43	4
Hexythiazox_1	Carboxamide acaricide/ insecticide	353	228	14.2	76	12	19	4
Hexythiazox_2		353	168.1	14.2	76	12	37	4
Hymexazol_1	Oxazole fungicide	100	54	1.26	56	10	21	10
Hymexazol_2		100	44	6.04	56	10	27	10
Imazamethabenz-methyl_1	Imidazolinone herbicide	289.1	144	7.96	71	11	45	6.5
Imazamethabenz-methyl_2		289.1	89.1	7.95	61	11	91	14
Imazamox_1	Imidazolinone herbicide	306.1	261.1	6.23	5	10	29	15
Imazamox_2		306.1	193	6.22	5	10	35	15
Imazapic_1	Imidazolinone herbicide	276.1	163.1	6.31	84	9.5	35	6.5
Imazapyr_1	Imidazolinone herbicide	262.2	217.2	12	81	7	23	4
Imazapyr_2		262.2	149.2	9	81	7	35	4
Imazaquin_1	Imidazolinone herbicide	312.2	267.2	10.9	86	5	25	4
Imazaquin_2		312.2	199.2	12.4	86	5	41	4
Imidacloprid_1	Neonicotinoid insecticide	256.2	209	4.83	71	7	23	4
Imidacloprid_2		256.2	175.2	4.83	71	7	23	4
Indoxacarb_1	Oxadiazine insecticide	528.1	203	13.4	71	10	51	6.5
Indoxacarb_2		528	56	13.4	71	10	55	4
Iodosulfuron-methyl_1	Sulfonylurea herbicide	507.9	167.2	10.5	79	9	27	6.5
Iodosulfuron-methyl_2		508	141	10.5	81	10	35	6
Iprobenfos_1	Organophosph ate fungicide	289.1	247.1	12.3	46	10	13	12
Iprobenfos_2		289.1	205.2	12.3	46	10	17	8
Iprodione_1	Dicarboximid e fungicide	330	143	11.8	106	10	21	6
Iprodione_2		330	101	11.8	81	11.5	33	6.5
Iprovalicarb_1	Carbamate fungicide	321.2	203.2	11.8	61	10	12	2
Iprovalicarb_2		321.2	119	11.8	89	12	23	6.5
Isoproturon_1	Phenylurea herbicide	207.1	165.2	9.46	86	10.5	19	6.5
Isoproturon_2		207.2	72.1	9.46	86	4	29	4
Isoxaflutole_1	Oxazole herbicide	259.9	184.1	13	50	10	45	8
Lenacil_1	Uracil herbicide	235.3	153.2	9.39	61	6.5	23	4
Lenacil_2		235.3	136.2	9.39	61	6.5	43	4
Linuron_1	Phenylurea herbicide	249	182.1	10.4	71	11	21	4
Linuron_2		249	160	10.4	71	11	23	4
Lufenuron_1	Benzoylurea insecticide/ acaricide	511.1	158.1	14.5	81	8	27	4
Lufenuron_2		511.1	141.2	14.5	81	8	67	4
Malaoxon_1	Metabolite	315	127.1	8.61	71	10.5	17	6.5
Malaoxon_2		315	99	8.61	71	10	31	4

Mandipropamid 1	Amide fungicide	412.1	328.1	11.3	80	10	22	4
Mandipropamid 2		412.1	125.1	11.3	80	10	70	4
Mecarbam_1	Organophosphate insecticide	330	227	11.9	59	9	15	6.5
Mecarbam_2		330	97	11.9	66	6.5	49	4
Mefenacet_1	Anilide herbicide	299.1	148.1	11.3	54	8	19	6.5
Mefenacet_2		299.2	120.1	11.3	36	8	35	6
Mefentrifluconazole_1	Triazole fungicide	398.2	70.2	12.4	128.2	10	60	6
Mefentrifluconazole_2		398.2	182	12.4	128.2	10	43	6
Melbmectin_1	Bio-insecticide	640	528.2	16	30	10	9	8
Melbmectin_2		640	498.3	16	28	10	17	8
Mesosulfuron methyl_2	Sulfonylurea herbicide	504	306	9.73	44	8.6	30.6	16.3
Mesosulfuron-methyl_1		504.1	182.1	9.73	81	12	33	6.5
Mesotrione_1	Aroylcyclohexanedione herbicide	357.1	227.8	6.91	36	7	29	12
Mesotrione_2		357.1	104.1	6.91	36	11.5	49	4
Metaflumizone_1	Semicarbazone insecticide	506.9	287	14.2	106	10	35	8
Metaflumizone_2		506.9	178	14.2	106	10	37	8
Metamitron_1	Triazinone herbicide	203.2	175.1	4.77	76	8.5	23	4
Metamitron_2		203.2	104.1	4.75	76	8.5	33	4
Metconazole_1	Triazole fungicide	320	125	12.6	81	10	49	6
Metconazole_2		320.1	70.1	12.6	76	12	45	6.5
Methabenzthiazuron_1	Urea herbicide	222.1	165.2	9.08	71	4	21	4
Methabenzthiazuron_2		222.1	150.3	9.09	71	4	41	4
Methacrifos_1	Organophosphate insecticide	241	209.1	9.37	71	10	13	6.5
Methacrifos_2		241	209	9.37	71	10	15	6.5
Methamidophos_1	Organophosphate insecticide	142	124.9	14.9	66	12	19	6.5
Methamidophos_2		142	94	0.87	71	5.5	19	6.5
Methiocarb_1	Carbamate insecticide	226.1	169.2	10.6	61	9	13	4
Methiocarb_2		226.1	121.1	10.6	61	9	23	4
Methiocarb-sulfone_1	Metabolite	258.1	201.2	6.23	68	10	13	2
Methiocarb-sulfone_2		258.1	122.1	6.22	68	10	25	3
Methiocarb-sulfoxide_1	Metabolite	242.1	185.1	5.5	74	8.5	19	10
Methiocarb-sulfoxide_2		242.1	122	5.51	74	8.5	39	6
Methomyl_1	Carbamate insecticide	163.1	106	2.85	56	7	13	4
Methomyl_2		163.1	88.1	2.83	56	7	13	4
Methoxyfenozide_1	Carbohydrazide insecticide	369.3	313.3	11.7	90	7	10	6
Methoxyfenozide_2		369.3	149.1	11.6	90	7	10	4
Metobromuron_1	Phenylurea herbicide	259	170	9.01	76	8.5	25	6.5
Metobromuron_2		259	148.2	9.01	76	7.5	21	4
Metoxuron_1	Phenylurea herbicide	229	156.1	6.92	76	9.5	29	4
Metoxuron_2		229	71.9	6.91	76	9.5	35	4
Metribuzin_1		215.1	187.2	7.57	81	7	21	4

Metribuzin_2	Triazinone herbicide	215.1	84.1	7.57	81	7	29	4
Metsulfuron-methyl_1	Sulfonylurea Herbicide	382.1	198.9	8.62	74	10.5	27	6.5
Metsulfuron-methyl_2		382	167	8.63	76	10	21	6
Mevinphos_1	Organophosphate insecticide	225	193.1	4.07	61	8.5	11	4
Mevinphos_2		225	127.1	4.04	61	8.5	19	4
Monocrotophos_1	Organophosphate insecticide	224.1	127	4.06	86	11.5	21	6.5
Monocrotophos_2		224	98	4.09	86	10	17	4
Monolinuron_1	Phenylurea herbicide	215	126	8.57	66	9	21	4
Monolinuron_2		215	99	8.57	66	9	43	4
Monuron_1	Phenylurea herbicide	199	72	7.35	71	11	27	4
Monuron_2		199	125.9	7.37	71	11	33	4
Moxidectin_1	Avermectin parasiticide	640.3	528.3	16	58	8	19	6
Moxidectin_2		640.3	199.2	16	56	8	31	4
Nicosulfuron_1	Sulfonylurea herbicide	411	213	8.2	101	10	23	7
Nicosulfuron_2		411.1	182.1	8.21	81	9	25	6.5
Nitenpyram_1	Neonicotinoid insecticide	271.1	225.1	1.93	66	8.5	15	4
Nitenpyram_2		271.1	126	1.96	66	8.5	37	4
Novaluron_1	Benzoylurea insecticide	510	158	13.9	51	11.5	31	6.5
Novaluron_2		493	141.1	13.9	90	10	69	4
Nuarimol_1	Pyrimidine fungicide	315	252.1	10.7	86	10	31	4
Nuarimol_2		315	81.1	10.7	86	10	39	4
Ofurace_1	Anilide fungicide	282.1	254	8.23	84	10.5	17	6.5
Ofurace_2		282	160	8.22	86	10	29	6
Omethoate_1	Organophosphate insecticide/ acaricide	214	124.9	1.5	66	8	29	6.5
Omethoate_2		214	182.8	1.58	70	10	18	4
Orthosulfamuron_1	Sulfonylurea herbicide	425	227	9.95	150	10	47	9
Orthosulfamuron_2		425	199	9.82	36	10	12	2
Oxadiargyl_1	Oxadiazolone herbicide	358	223	12.9	74	9.5	27	6.5
Oxadiargyl_2		340.8	151	12.9	81	10	33	10
Oxadiazon_1	Oxadiazolone herbicide	362.1	303.1	14	79	10	22	6.5
Oxadiazon_2		362.1	220	14	79	8	31	6.5
Oxadixyl_1	Anilide fungicide	279.2	219.2	7.43	66	8.5	15	4
Oxadixyl_2		279.2	132.1	7.43	66	8.5	41	4
Oxamyl_1	Carbamate insecticide	237.1	90.1	2.68	63	4	11	3
Oxamyl_2		237.1	72	2.64	55	5.5	21	6.5
Oxasulfuron_1	Sulfonylurea herbicide	407.1	150.1	8.28	71	9	25	6.5
Oxasulfuron_2		407.1	107	8.28	56	10	83	12
Oxycarboxin_1	Oxathiin fungicide	268	175.1	5.8	76	11.5	19	6.5
Oxycarboxin_2		268	147	5.81	76	10	29	6
Paclobutrazol_1		296.2	70.1	10.5	117	10	49	10

Paclobutrazol_2	Triazole fungicide	296.2	70	10.5	144	10	53	10
Paraoxon-methyl_1	Organophosphate insecticide	248	202	7.28	96	10	25	7
Paraoxon-methyl_2		248	109.1	7.28	74	11	35	6.5
Pencycuron_1	Phenylurea fungicide	329.1	125.1	13	86	11	33	6.5
Pencycuron_2		329	99	13	91	10	83	4
Penflufen_1	Anilide fungicide	318	234	12.3	144	6	24	11
Penflufen_2		318	141.1	12.3	144	6	39	15
Penoxsulam_1	Triazolopyrimidine herbicide	484	195	9.42	48.3	6	38	9.8
Penoxsulam_2		484	164	9.43	48.3	6	44.9	8.6
Penthiopyrad_1	Carboxamide fungicide	360	276	12.5	121	10	26	8
Penthiopyrad_2		360	177	12.5	121	10	63	8
Phenmedipham_1	Carbanilate herbicide	301.1	168.1	10.4	70	10	14	4
Phenmedipham_2		301.1	136.1	10.4	76	12	25	6.5
Phosmet_1	Organophosphorus insecticide/ acaricide	318	160	10.5	66	10	19	6
Phosmet_2		317.9	133.1	10.5	69	9.5	49	6.5
Phosphamidon_1	Organophosphate insecticide	300	174	7.76	71	10	19	7
Phosphamidon_2		300	127.1	7.75	74	11	27	6.5
Phoxim_1	Organophosphate insecticide	299	129.1	12.9	66	9	17	6.5
Phoxim_2		299	77	12.9	66	10	41	4
Picarbutrazox_1	Carbamate fungicide	410.2	310.2	12.04	61	10	19	12
Picarbutrazox_2		410.2	106.9	12.04	61	10	33	12
Picoxystrobin_1	Strobilurin fungicide	368	205	12.4	81	10	15	7
Picoxystrobin_2		368.1	145	12.4	79	6.5	27	6.5
Pinoxaden 1	Phenylpyrazole herbicide	401	317	12.9	25	5.8	31	8
Pinoxaden 2		401	57	12.9	25	5.8	63	10
Pirimicarb_1	Carbamate insecticide	239.1	72.1	6.19	56	7	31	6.5
Pirimicarb_2		239.2	182.2	6.19	73	10	21	2
Praziquantel 1	Anthelmintic	313.1	203.3	10.8	33	10	23	13
Pretilachlor_1	Chloroacetanilide herbicide	312	252	13.4	81	10	23	26
Pretilachlor_2		312	176	13.4	81	10	39	24
Prochloraz_1	Amide fungicide	376	308.1	12.4	56	7.5	17	6
Prochloraz_2		376	69.9	12.4	56	7.5	43	4
Profenofos_1	Organophosphate insecticide	372.9	302.9	13.5	86	10	25	6.5
Profenofos_2		373	97	13.5	76	10	43	4
Promecarb_1	Carbamate insecticide	208.2	109.1	10.8	61	9.5	23	4
Promecarb_2		208.2	151.1	10.8	61	9.5	13	4
Prometryne_1	Triazine herbicide	242.2	158.1	10.2	91	10	35	4
Prometryne_2		242.2	200.1	10.2	91	10	19	4
Propachlor_1	Chloroacetanilide herbicide	212.2	170.1	9.51	71	7	19	4
Propachlor_2		212.2	94.1	9.5	71	7	35	4

Propamocarb_1	Carbamate fungicide	189.2	102	1.26	56	10	23	6.5
Propamocarb_2		189.2	73.9	0.94	71	10	35	3
Propanil_1	Anilide herbicide	218.1	162.1	10.3	76	8.5	21	4
Propanil_2		218.1	127.1	10.3	76	8.5	37	4
Propaquizafop_1	Aryloxyphenoxypropionate herbicide	444.1	299.1	13.8	99	9.5	31	6.5
Propaquizafop_2		444.1	100.1	13.8	99	9.5	30	6.5
Propargite_1	Sulfite ester insecticide/ acaricide	368.2	231.2	14.6	56	7	15	4
Propargite_2		368.2	175.1	14.6	56	7	21	4
Propazine_1	Triazine herbicide	230.2	188.3	10.5	91	4	23	4
Propazine_2		230.1	146	10.5	74	12	29	6.5
Propetamphos_1	Organophosphate insecticide/ acaricide	282.1	156.2	11.5	74	10	17	6.5
Propetamphos_2		282	138	11.5	76	10	21	6
Propyzamide_1	Amide herbicide	256	173.1	11	79	12	31	6.5
Propyzamide_2		256	190	11	81	10	19	7
Proquinazid_1	Quinazoline fungicide	373.2	331.1	14.6	46	10	21	20
Proquinazid_2		373.2	288.9	14.6	46	10	37	16
Prosulfocarb_1	Carbamate herbicide	252.3	91.1	13.4	71	4	35	4
Prosulfocarb_2		252.3	128.1	13.4	71	4	17	4
Prothioconazole_1	Conazole fungicide	344	326	12.3	64	9.5	21	6.5
Prothioconazole_2		344	125	12.3	61	9.5	15	6.5
Pydiflumetofen_1	Pyrazole fungicide	426.4	193.2	12.9	70	10	44	4.2
Pydiflumetofen_2		426.4	166.4	12.9	70	10	39	8.5
Pymetrozin_1	Pyridine insecticide	218	105	0.88	96	10	27	6
Pymetrozin_2		218	79	1.33	91	10	47	4
Pyraclostrobin_1	Strobilurin fungicide	388.1	194	12.8	59	7	19	6.5
Pyraclostrobin_2		388	163	12.8	51	10	29	7
Pyraflufen-ethyl_1	Phenylpyrazole herbicide	413	339	12.7	104	11.5	25	6.5
Pyraflufen-ethyl_2		413	252.9	12.7	104	11.5	50	6.5
Pyrazosulfuron-ethyl_1	Sulfonylurea herbicide	415	182	11.6	96	10	25	10
Pyrazosulfuron-ethyl_2		415	139	11.6	101	10	61	14
Pyridalyl _1	insecticide	490	183	16.3	32.6	7.8	22.9	5.4
Pyridalyl _2		490	109	16.3	32.6	7.8	29.8	11.27
Pyrifenox_1	Pyridine fungicide	295	263	10.7	50	12	35	10
Pyrifenox_2		295	93.1	10.7	56	12	31	6.5
Pyroxsulam_1	Pyridine herbicide	435	195	8.48	96	10	39	6
Pyroxsulam_2		435	124	8.48	96	10	65	6
Quinclorac_1	Quinolinecarboxylic acid herbicide	241.9	223.9	6.85	20	10	23	15
Quinclorac_2		241.9	161	6.85	20	10	53	15
Quinmerac_1	Quinolinecarboxylic acid herbicide	222	204.1	5.02	64	10	23	6.5
Quinmerac_2		222	141	5.01	66	10	43	6
Quizalofop-ethyl_1		373.1	298.9	13.6	94	11.5	25	6.5

Quizalofop-ethyl_2	Aryloxyphenoxypropionate herbicide	373	271	13.6	116	10	33	10
Quizalofop-P (free acid)_1	Aryloxyphenoxypropionate herbicide	345.1	299.1	13.6	104	12	25	6.5
Rimsulfuron_1	Sulfonylurea herbicide	432.1	182	9.05	71	12	29	6.5
Rimsulfuron_2		432	325	9.05	96	10	21	12
Rotenone_1	Botanical insecticide	395.1	213.2	12.1	96	9.5	29	4
Rotenone_2		395.1	192.1	12.1	96	9.5	31	4
Sebuthylazine_1	Triazine herbicide	230.1	174.2	10.8	66	11.5	25	6.5
Sebuthylazine_2		230.2	104.1	10.8	81	5.5	41	4
Sebuthylazine-desethyl_1	Triazine herbicide	202.1	145.9	7.34	71	10.5	25	6.5
Sebuthylazine-desethyl_2		202.1	79.1	7.33	61	10	37	10
Spinetoram A 1	Spinosyn insecticide	748.5	142.2	12.9	95	10	50	4
Spinetoram A 2		748.5	98.1	12.8	95	10	109	4
Spinetoram B 1	Spinosyn insecticide	760.5	142.2	13.2	105	10	50	4
Spinetoram B 2		760.5	98.1	13.2	105	10	110	4
Spinosyn A_1	Spinosyn insecticide	732.4	142.2	12.3	86	9.5	41	2
Spinosyn A_2		732.4	98.2	12.3	86	9.5	93	2
Spinosyn D_1	Spinosyn insecticide	746.4	142.2	12.7	86	10	41	2
Spinosyn D_2		746.4	98.2	12.7	86	10	105	2
Spirotetramat_1	Tetramic acid insecticide	374.4	330.2	11.7	74.9	10	21.2	4
Spirotetramat_2		374.4	302.4	11.7	74.9	12	23.6	6.5
Spiroxamine_1	Morpholine fungicide	298.4	144.2	9.83	81	5	27	4
Spiroxamine_2		298.4	100.2	9.83	81	5	43	4
Sulcotrione_1	Aroylcyclohexanedione herbicide	329	111	7.74	86	10	39	10
Sulcotrione_2		346	139	7.74	81	10	31	6.5
Sulfometuron-methyl_1	Sulfonylurea herbicide	365.1	150.1	8.58	74	9.5	23	6.5
Sulfometuron-methyl_2		365.1	107.1	8.58	74	10.5	29	8
Sulfoxaflor_1	Sulfoximine insecticide	278	174	5.95	37	8.4	17	10
Sulfoxaflor_2		278	105	5.95	37	8.4	26	10
Tebufenozide_1	Carbohydrazide insecticide	353.2	133.2	12.3	61	8.5	23	4
Tebufenozide_2		353.2	297.2	12.3	61	8.5	15	4
Tebuthiuron_1	Urea herbicide	229.1	172.2	8.19	64	10	23	6.5
Tebuthiuron_2		229.2	116.1	8.19	66	3.5	35	4
Teflubenzuron_1	Phenylurea insecticide	381.2	118	15.6	75	10	31	9
Temephos_1	Organophosphate insecticide	484	125	9.44	51	8.5	49	6.5
TEPP_1	Organophosphate insecticide	291.1	179	7.78	71	10.5	27	6.5
TEPP_2		291.1	99	7.78	71	10.5	49	6.5
Tepraloxydim_1	Cyclohexene oxime herbicide	342	250	11.6	46	10	19	24
Tepraloxydim_2		342	166	11.5	46	10	31	12

Terbumeton_1	Triazine herbicide	226.1	170.2	8.14	56	11.5	23	6.5
Terbumeton_2		226	114	8.12	56	10	35	6
Tetraconazole_1	Triazole fungicide	372	159	11.8	81	10	39	6.5
Tetraconazole_2		372	70	11.8	76	10	47	4
Thiabendazole_1	Benzimidazole fungicide	202.1	175.1	3.66	96	5	35	4
Thiabendazole_2		202.1	131.2	3.76	96	5	43	4
Thiacloprid_1	Neonicotinoid insecticide	253.1	126.1	6.44	81	8.5	29	4
Thiacloprid_2		253.1	99.1	6.45	81	8.5	57	4
Thiamethoxam_1	Neonicotinoid insecticide	292	211	3.53	74	8.5	17	6.5
Thiamethoxam_2		292	181	3.54	96	10	31	7
Thifensulfuron-methyl_1	Sulfonylurea herbicide	388	167	8.34	69	9	21	6.5
Thifensulfuron-methyl_2		388	205	8.34	61	10	33	7
Thiocyclam-OH_1	2-dimethylaminopropane-1,3-dithiol analogue insecticide	182	137	6.21	46	10	23	10
Thiocyclam-OH_1		182	73	6.21	46	10	31	10
Thiodicarb_1	Carbamate insecticide	355	88	9.35	66	10	21	6.5
Thiodicarb_2		355.1	108	9.35	60	10	21	3
Thiofanox_1	Carbamate insecticide	241	184	6.21	76	10	17	18
Thiofanox_2		241	98	4.05	76	10	19	12
Thiophanate-methyl_1	Carbamate fungicide	343	151	8.04	66	10.5	25	6.5
Thiophanate-methyl_2		343	192	8.04	66	10	21	7
Thiram_1	Dithiocarbamate fungicide	241	88	9.31	61	10	19	3
Thiram_2		241	120	8.04	61	10	20	3
Tolfenpyrad 1	Pyrazole insecticide	384	145	13.9	121	10	39	10
Tolfenpyrad 2		384	154	13.9	121	10	59	10
Tolyfluanid_1	Sulfamide fungicide	364	237.9	12.5	6	10	19	10
Tolyfluanid_2		364	137.1	12.5	6	10	37	10
Tralkoxydim_1	Cyclohexene oxime herbicide	330.2	138	14.3	89	10	27	6.5
Tralkoxydim_2		330.3	284.2	14.3	117	10	17	18
Triasulfuron_1	Sulfonylurea herbicide	402.1	167.1	8.41	84	10	25	6.5
Triasulfuron_2		402	141	8.41	81	10	29	6
Triazophos_1	Organophosphate insecticide/ acaricide	314	162	11.5	76	2	25	6.5
Triazophos_2		314	119.1	11.5	71	12	47	6.5
Trichlorfon_1	Organophosphate insecticide/ acaricide	274	221	4.71	51	10	21	7
Trichlorfon_2		274	108.9	4.71	59	8.5	31	6.5
Triclopyr-2-Butotyl_1	Pyridine herbicide	356.2	237.7	13.8	66	10	15	14
Triclopyr-2-Butotyl_2		356.2	281.9	13.8	66	10	17	18
Tricyclazole_1	Triazolobenzothiazole fungicide	190	163.1	6.62	89	12	31	6.5
Tricyclazole_2		190	136	6.62	91	10	37	6

Trifloxystrobin_1	Strobilurin fungicide	409.1	186.1	13.5	59	7.5	23	6.5
Trifloxystrobin_2		409	206	13.5	46	10	21	7
Triflumizole_1	Conazole fungicide	346.1	278.1	13.2	56	7	15	4
Triflumizole_2		346.1	72.9	13.2	56	7	23	4
Triflumuron 1	Benzoylurea insecticide	359.1	156.2	12.9	70	10	23	4
Triflumuron 2		359.1	139	12.9	70	10	43	4
Triflusulfuron methyl_1	Sulfonylurea herbicide	493	264	11.4	76	10	29	14
Triflusulfuron methyl_2		493	96	11.4	34	10	45	4
Triforin_1	Amide fungicide	434.9	389.8	10.4	56	10	17	8
Triforin_2		434.9	215.1	10.3	56	10	37	20
Triticonazole_1	Triazole fungicide	318.1	70	11.5	66	9.5	29	4
Triticonazole_2		318.1	124.9	11.5	66	9.5	49	4
Zoxamide_1	Benzamide fungicide	336.1	186.9	12.5	85	10	30	4
Zoxamide_2		338.1	188.7	12.5	85	10	35	4

* Quantifier ions

** Qualifier ions

Table S2: GC-MS/MS optimization parameters including retention time (RT), molecular masses to charge ratio (m/z) in the quads (q1&q3), collision energy (CE) for all target MRM transitions of GC-Compounds.

Pesticide Name	Chemical class and type	RT	Q1 *(m/z)*	Q3 (m/z)	CE (Volts)
Acetochlor_1	Chloroacetamide herbicide	7.84	222.9	132.2	20
Acetochlor_1		7.84	222.9	147.2	5
Aclonifen_1	Diphenyl ether herbicide	9.71	264	194.1	14
Aclonifen_2		9.71	264	212.1	12
Alachlor_1	Chloroacetamide herbicide	7.94	188.1	130	32
Alachlor_2		7.94	188.1	160.1	8
Aldrin_1	Organochlorine insecticide	8.36	262.7	191	30
Aldrin_2		8.36	262.7	192.9	32
Ametryne_1	1,3,5-triazine herbicide	7.93	227.1	58.1	12
Ametryne_2		7.93	227.1	170	10
Amisulbrom_1	Sulfonamide fungicide	11.72	225.9	120	25
Amisulbrom_2		11.72	225.9	147	15
Amitraz_1	Amidine acaricide and insecticide	9.95	132.1	90	35
Amitraz_2		9.95	132.1	117.1	15
Anilofos_1	Organophosphate herbicide	10.69	225.9	157	14
Anilofos_2		10.69	225.9	184	6
Atrazine_1	Triazine herbicide	7.06	200	122.1	8
Atrazine_2		7.06	215.1	58.1	12
Azaconazole_1	Triazole fungicide	9.42	217	172.9	14
Azaconazole_2		9.42	218.9	174.9	14
Azinphos-ethyl_1	Organophosphorus insecticide and acaricide	11.2	132	77	12
Azinphos-ethyl_2		11.2	160	77	16
Azinphos-methyl_1	Organophosphorus insecticide	10.52	132	77	12
Azinphos-methyl_2		10.52	160	50.9	34
Benalaxyl_1	Phenylamide (acylalanine) fungicide	9.96	233.9	146	20
Benalaxyl_2		9.96	266	148.1	5
Bifenazate_1	Acaricide	10.51	258	196.1	12
Bifenazate_2		10.51	258	199.1	12
Bifenthrin_1	Pyrethroid insecticide and acaricide	10.48	181	165.9	10
Bifenthrin_2		10.48	181	179	12
Bitertanol_1	Triazole fungicide	11.35	170	115.1	34
Bitertanol_2		11.35	170	141.1	20
Bromuconazole_1	Triazole fungicide	10.49	172.9	109	26
Bromuconazole_2		10.49	172.9	144.9	14
Bupirimate_1	Pyrimidinol fungicide	9.38	208.1	140.1	12
Bupirimate_2		9.38	273.1	193.2	8

Buprofezin_1	Thiadiazine insecticide	9.37	105	104.1	10
Buprofezin_2		9.37	175	117	25
Butralin_1	Dinitroaniline herbicide	8.51	266.1	190.1	10
Butralin_2		8.51	266.1	219.9	10
Cadusafos_1	Organophosphorus nematicide and insecticide	6.73	159	96.9	16
Cadusafos_2		6.73	213	89.1	12
Captan_1	Phthalimide fungicide	8.81	116.9	82	30
Captan_2		8.81	149	69.9	20
Carfentrazone-ethyl_1	Triazolinone herbicide	9.91	290	99.9	36
Carfentrazone-ethyl_2		9.91	340.1	312.1	10
Chlorfenapyr_1	Insecticide and acaricide	9.51	248.9	112	24
Chlorfenapyr_2		9.51	248.9	137.1	18
Chlorfenvinphos_1	Organophosphorus insecticide and acaricide	8.75	266.9	159	16
Chlorfenvinphos_2		8.75	266.9	203	10
Chlorothalonil_1	Chloronitrile fungicide	7.53	263.8	133	40
Chlorothalonil_2		7.53	265.8	133.1	36
Chlorpropham_1	Carbamate herbicide	6.5	171	127	8
Chlorpropham_2		6.5	213	127	14
Chlorpyrifos-ethyl_1	Organophosphorus insecticide	8.35	196.7	107	36
Chlorpyrifos-ethyl_2		8.35	313.9	257.9	12
Chlorpyrifos-methyl_1	Organophosphorus insecticide and acaricide	7.87	285.9	92.9	20
Chlorpyrifos-methyl_2		7.87	287.9	272.7	15
Chlorthiophos_1	Organophosphate insecticide	9.74	296.9	268.9	8
Chlorthiophos_2		9.74	324.9	269	12
Clodinafop-propargyl_1	Aryloxyphenoxypropionate herbicide	10	266	91.1	14
Clodinafop-propargyl_2		10	349.1	266.1	8
Clomazone_1	Isoxazolidinone herbicide	7.13	125	89	16
Clomazone_2		7.13	138	74.9	24
Cyflufenamid_1	Amidoxine fungicide	9.46	222.8	203	10
Cyflufenamid_2		9.46	237	188	24
Cyfluthrin_1	Pyrethroid insecticide	11.76	198.9	170.1	25
Cyfluthrin_2		11.76	206	150	40
Cyhalothrin I (lambda)_1	Pyrethroid insecticide	10.97	197.1	141.1	10
Cyhalothrin I (lambda)_2		10.97	207.9	180.9	5
Cypermethrin_1	Pyrethroid insecticide	11.93	163	91.1	12
Cypermethrin_2		11.93	163	127.1	6
Cyproconazole_1	Triazole fungicide	9.53	222	82.1	10
Cyproconazole_2		9.53	222	125	20
Cyprodinil_1	Anilinopyrimidine fungicide	8.61	224.1	196.9	20
Cyprodinil_2		8.61	224.1	208	18
p,p-DDD_1	Organochlorine insecticide	9.7	235	165.1	20

p,p-DDD_2		9.7	236.8	165	20
O,p-DDD_1	Organochlorine insecticide	9.39	235	165.1	20
O,p-DDD_2		9.39	236.8	165	20
P,p-DDE_1	Organochlorine insecticide	9.3	246	176.1	28
P,p-DDE_2		9.3	317.8	246	20
P,p-DDT_1	Organochlorine insecticide	10.05	235	165.1	20
P,p-DDT_2		10.05	236.8	165	20
Deltamethrin_1	Pyrethroid insecticide	12.9	252.8	92.9	16
Deltamethrin_2		12.9	252.8	172	8
Diazinon_1	Organophosphorus insecticide and acaricide	7.33	137.1	54.1	20
Diazinon_2		7.33	137.1	84.1	12
Dichlofenthion_1	Organophosphate insecticide	7.76	222.9	205	12
Dichlofenthion_2		7.76	250.9	223	8
dichlorvos_1	Organophosphorus insecticide and acaricide	3.96	184.9	93	10
dichlorvos_2		3.96	184.9	109	15
Diclofop methyl_1	Aryloxyphenoxypropionate herbicide	10.16	252.9	161.9	16
Diclofop methyl_2		10.16	340	253	10
Diethofencarb	N-phenyl carbamate fungicide	8.24	225.1	96	24
Difenoconazole_1	Triazole fungicide	12.69	265	139	36
Difenoconazole_2		12.69	323	265	16
Diflufenican_1	Pyridinecarboxamide herbicide	10.16	266	246.1	10
Diflufenican_2		10.16	394	266.1	12
Dimethachlor_1	Chloroacetamide herbicide	7.78	134	105.1	12
Dimethachlor_2		7.78	197	148.1	10
Dimethenamid_1	Chloroacetamide herbicide	7.8	154.1	137	8
Dimethenamid_2		7.8	230	154.1	10
Dimethomorph_1	Cinnamic acid fungicide	13.37	301	165.1	10
Dimethomorph_2		13.37	387.1	301.1	10
Diniconazole_1	Triazole fungicide	9.67	268	136	34
Diniconazole_2		9.67	268	232	10
Diphenylamine_1	Fungicide	6.37	168.1	167.1	14
Diphenylamine_2		6.37	169.2	167.1	22
Dodemorph_1	Morpholine fungicide	8.52	154.1	81.9	18
Dodemorph_2		8.52	154.1	96.6	10
Edifenphos_1	Phosphorothiolate fungicide	10	201	109	10
Edifenphos_1		10	309.9	108.9	25
Endosulfan_1	Organochlorine insecticide and acaricide	9.12	194.7	125	22
Endosulfan_2		9.12	240.6	205.9	14
Endrin_1	Organochlorine insecticide	9.59	245	173	22
Endrin_2		9.59	262.8	192.9	30

EPN_1	Organophosphorus insecticide and acaricide	10.52	157	77	22
EPN_2		10.52	169	77	22
Epoxiconazole_1	Triazole fungicide	10.34	192	111	22
Epoxiconazole_2		10.34	192	138	12
Esfenvalerate_1	Pyrethroid insecticide	12.53	167	125	10
Esfenvalerate_2		12.53	225.1	119.1	18
Ethion_1	Organophosphorus insecticide and acaricide	9.71	230.9	128.9	22
Ethion_2		9.71	230.9	174.9	12
Ethoprophos_1	Organophosphorus nematicide and insecticide	6.42	157.9	97	15
Ethoprophos_2		6.42	199.9	97	20
Etofenprox_1	Pyrethroid insecticide	12	163.1	107.1	16
Etofenprox-2		12	163.1	135.1	10
Etrimfos_1	Insecticide	7.5	153.1	56	16
Etrimfos_2		7.5	153.1	98	10
Fenamidone_1	Imidazole fungicide	10.63	238.1	103.1	22
Fenamidone_2		10.63	268	180.1	18
Fenamiphos_1	Organophosphorous insecticide and nematicide	9.12	154	139	10
Fenamiphos_2		9.12	303.1	195.2	8
Fenarimol_1	Pyrimidine fungicide	11.14	139	74.9	26
Fenarimol_2		11.14	139	111	14
Fenazaquin_1	Quinazoline acaricide	10.65	145.1	117.1	12
Fenazaquin_2		10.65	160.1	145.1	8
Fenbuconazole_1	Triazole fungicide	11.71	129	102	14
Fenbuconazole_2		11.71	198.1	129.1	8
Fenhexamid_1	Hydroxyanilide fungicide	10.06	177.1	113	15
Fenhexamid_2		10.06	301	97	15
Fenitrothion_1	Organophosphorus insecticide	8.15	277	109	16
Fenitrothion_2		8.15	277	260	6
fenpropathrin_1	Pyrethroid acaricide and insecticide	10.55	207.9	181	5
fenpropathrin_2		10.55	264.9	210	10
Fenpropimorph_1	Morpholine fungicide	8.32	128.1	70.1	12
Fenpropimorph_2		8.32	128.1	110.1	8
Fenthion_1	Organophosphorus insecticide	8.34	278	109	18
Fenthion_2		8.34	278	169	14
Fenvalerate_1	Pyrethroid insecticide and acaricide	12.4	167	89	32
Fenvalerate_2		12.4	167	125	10
Fipronil_1	Phenylpyrazole insecticide	8.74	366.9	212.9	28
Fipronil_2		8.74	366.9	244.9	20
Fluazifop-P-butyl_1	Aryloxyphenoxypropionate herbicide	9.46	282	91.1	18
Fluazifop-P-butyl_2		9.46	282	238.1	16
Flucythrinate_1	Pyrethroid insecticide	11.95	157	107.1	12

Flucythrinate_2		11.95	199.1	107.1	22
Fludioxonil_1	Phenylpyrrole fungicide	9.23	248	127	26
Fludioxonil_2		9.23	248	153.8	18
Flumioxazin_1	N-phenylphthalimide herbicide	12.42	354.1	312	8
Flumioxazin_2		12.42	354.1	326.1	8
Fluroxypyr meptyl_1	Pyridinecarboxylic acid herbicide	10.17	180.9	134	20
Fluroxypyr meptyl_2		10.17	180.9	161	10
Flusilazole_1	Triazole fungicide	9.37	233	151.9	14
Flusilazole_2		9.37	233	164.9	16
Flutolanil_1	Oxathiin fungicide	9.15	173	95	28
Flutolanil_2		9.15	281	173	10
Flutriafol_1	Triazole fungicide	9.13	123	75	24
Flutriafol_2		9.13	219	123	12
Folpet_1	Phthalimide fungicide	8.87	259.8	130.1	15
Folpet_2		8.87	261.8	130.1	15
Haloxyfop-methyl_1	Aryloxyphenoxypropionate herbicide	8.89	179.9	160	10
Haloxyfop-methyl_2		8.89	375.2	316	10
Heptachlor_1	Organochlorine insecticide	7.99	99.8	65	12
Heptachlor_2		7.99	271.8	236.9	12
Hexachlorobenzene_1	Organochlorine fungicide	6.97	283.8	248.8	18
Hexachlorobenzene_2		6.97	285.8	250.8	18
Hexazinone_1	1,2,4-triazinone herbicide	10.14	171.1	71.1	14
Hexazinone_2		10.14	171.1	85.1	12
Imazalil_1	Imidazole fungicide	9.23	172.8	109	26
Imazalil_2		9.23	215	173	8
Iprobenfos_1	Phosphorothiolate fungicide	7.59	203.9	91.1	8
Iprobenfos_2		7.59	203.9	121	28
Iprovalicarb_1	Carbamate fungicide	9.27	118.9	117.1	8
Iprovalicarb_2		9.27	134.1	42	20
Isoprothiolane_1	Phosphorothiolate fungicide	9.22	204	118	8
Isoprothiolane_2		9.22	290	118	12
Kresoxim-methyl_1	Oximinoacetate fungicide	9.38	116	62.9	24
Kresoxim-methyl_2		9.38	116	89	14
Malathion_1	Organophosphorus insecticide and acaricide	8.22	126.9	99	5
Malathion_2		8.22	172.9	99	15
Mefenpyr-Diethyl_1	Herbicide safener	10.32	253	190	20
Mefenpyr-Diethyl_2		10.32	299	252.9	10
Mepanipyrim_1	Anilinopyrimidine fungicide	9.04	222	206	26
Mepanipyrim_2		9.04	222	207.1	14
Mepronil_1	Oxathiin fungicide	9.78	119.1	65.1	25
Mepronil_2		9.78	269	119.1	15

Metalaxyl_1	Phenylamide fungicide	7.99	206.1	132.1	20
Metalaxyl_2		7.99	234	146.1	20
Metazachlor_1	Chloroacetamide herbicide	8.69	133.1	117.3	22
Metazachlor_2		8.69	209	132.1	16
Methidathion_1	Organophosphorus insecticide and acaricide	8.95	145	58	14
Methidathion_2		8.95	145	85	6
Methoxychlor_1	Organochlorine insecticide	10.56	227.1	141.1	32
Methoxychlor_2		10.56	227.1	169.1	22
Metolachlor_1	Chloroacetamide herbicide	8.33	238.1	132.8	26
Metolachlor_2		8.33	238.1	162.2	10
Metrafenone_1	Fungicide	11.24	393	346.9	20
Metrafenone_2		11.24	393	362.7	16
Metribuzin_1	1,2,4-triazinone herbicide	7.79	198	82.1	16
Metribuzin_2		7.79	198	110	10
Myclobutanil_1	Triazole fungicide	9.35	179	90	28
Myclobutanil_2		9.35	179	125	14
Napropamide_1	Alkanamide herbicide	9.18	100.1	72.1	8
Napropamide_2		9.18	128.2	72.1	6
Nuarimol_1	Pyrimidine fungicide	10.2	235	123.1	15
Nuarimol_2		10.2	235	139.1	15
Ortho-phenylphenol_1	Fungicide	5.74	141.1	115.1	14
Ortho-phenylphenol_2		5.74	170.1	115	34
Oxadiazon_1	Oxadiazole herbicide	9.29	174.9	112	15
Oxadiazon_2		9.29	257.8	175.1	5
Oxadixyl_1	Phenylamide fungicide	9.73	163.1	117	24
Oxadixyl_2		9.73	163.1	132.1	8
Oxyfluorfen_1	Diphenyl ether herbicide	9.32	252	146	30
Oxyfluorfen_2		9.32	252	224	10
Parathion_1	Organophosphorus insecticide and acaricide	8.37	290.9	80.9	25
Parathion_2		8.37	290.9	109	10
Penconazole_1	Triazole fungicide	8.7	248	157	22
Penconazole_2		8.7	248	192	12
Pendimethalin_1	Dinitroaniline herbicide	8.68	252.1	161	14
Pendimethalin_2		8.68	252.1	162	8
Permethrin_1	Pyrethroid insecticide	11.37	183.1	153	12
Permethrin_2		11.37	183.1	168	12
Phenthoate_1	Organophosphorus insecticide and acaricide	8.79	246	121	8
Phenthoate_2		8.79	274	121	10
Phorate_1	Organophosphorus insecticide and acaricide	6.79	230.9	128.9	25
Phorate_2		6.79	260	75	5
Phosalone_1		10.86	182	74.8	30

Phosalone_2	Organophosphorus insecticide and acaricide	10.86	182	111	14
Picolinafen_1	Pyridinecarboxamide herbicide	10.51	238	145.1	22
Picolinafen_2		10.51	376.1	238.7	12
Picoxystrobin_1	Strobilurin fungicide	9.09	145.1	102.1	25
Picoxystrobin_2		9.09	145.1	115.1	15
Piperonyl butoxide_1	Insecticide synergist	10.19	176.1	103.1	22
Piperonyl butoxide_2		10.19	176.1	131.1	12
Pirimicarb_1	Carbamate insecticide	7.62	166.1	55	18
Pirimicarb_2		7.62	238.1	166.1	10
Pirimiphos-ethyl_1	Organophosphorus insecticide	8.54	304	168.1	12
Pirimiphos-ethyl_2		8.54	318.1	166.1	12
Pirimiphos-methyl_1	Organophosphorus insecticide and acaricide	8.14	290.1	125	20
Pirimiphos-methyl_2		8.14	290.1	233	8
Pretilachlor_1	Chloroacetamide herbicide	9.26	202.1	174.2	8
Pretilachlor_2		9.26	238.1	146.1	10
Prodiamine_1	Dinitroaniline herbicide	8.11	275.1	255.1	8
Prodiamine_2		8.11	321.1	279.1	6
Profenofos_1	Organophosphorus insecticide and acaricide	9.25	336.9	266.9	12
Profenofos_2		9.25	338.8	268.7	15
Propazine_1	1,3,5-triazine herbicide	7.11	214.2	172.2	10
Propazine_2		7.11	229.1	58.1	10
Propiconazole_1	Triazole fungicide	10	172.9	109	26
Propiconazole_2		10	172.9	145	14
Propoxur_1	Carbamate insecticide	6.28	110	62.9	24
Propoxur_2		6.28	152.1	110	8
Propyzamide_1	Benzamide herbicide	7.26	172.9	109	26
Propyzamide_2		7.26	172.9	145	14
Prosulfocarb_1	Thiocarbamate herbicide	8.03	128.1	43.1	10
Prosulfocarb_2		8.03	251.1	128.1	5
Prothiofos_1	Organophosphorus insecticide	9.22	266.7	238.9	8
Prothiofos_2		9.22	308.9	239	14
Pyraflufen-ethyl_1	Phenylpyrazole herbicide	10.01	349	238	16
Pyraflufen-ethyl_2		10.01	349	266.1	10
Pyrazophos_1	Phosphorothiolate fungicide	11.12	221	148.7	14
Pyrazophos_2		11.12	221	193.1	8
Pyridaben_1	Pyridazinone insecticide	11.47	147.1	117.1	20
Pyridaben_2		11.47	147.1	132.1	12
Pyridalyl_1	Pyridalyl insecticide	12	163.8	146.1	12
Pyridalyl_2		12	204	148.1	18
Pyridaphenthion_1		10.44	199	92.1	14

Pyridaphenthion_2	Organophosphorus insecticide and acaricide	10.44	340	199.1	8
Pyrifenox-E_1	Pyridine fungicide	8.72	227	192.1	12
Pyrifenox-E_2		8.72	227	200	12
Pyrifenox-Z_1	Pyridine fungicide	8.98	262	200	14
Pyrifenox-Z_2		8.98	262	227	10
Pyrimethanil_1	Anilinopyrimidine fungicide	7.33	198.1	117.9	30
Pyrimethanil_2		7.33	198.1	182.9	14
Pyriproxyfen_1	Juvenile hormone mimic insecticide	10.86	136.1	96	10
Pyriproxyfen_2		10.86	226.1	186.1	12
Quinalphos_1	Organophosphorus insecticide and acaricide	8.8	146	118.1	8
Quinalphos_2		8.8	157.1	102	22
Quinoxyfen_1	Quinoline fungicide	10	271.9	237.1	10
Quinoxyfen_2		10	306.8	237	20
Simazine_1	1,3,5-triazine herbicide	7.01	201.1	138.1	10
Simazine_2		7.01	203.1	138.1	10
Spirodiclofen_1	Tetronic acid acaricide and insecticide	11.42	156.9	86.7	32
Spirodiclofen_2		11.42	312.2	259	8
Spiroxamine_1	Morpholine fungicide	7.84	100.1	58	10
Spiroxamine_2		7.84	100.1	72.1	8
Sulfotep_1	Organophosphorus insecticide and acaricide	6.7	202	145.9	10
Sulfotep_2		6.7	322	202	10
Sulprofos_1	Organophosphorus insecticide	9.84	156	108	30
Sulprofos_2		9.84	322	156.1	10
Tebuconazole_1	Triazole fungicide	10.18	125	89	16
Tebuconazole_2		10.18	250	125	20
Tebufenpyrad_1	Pyrazole acaricide	10.58	276.1	171	10
Tebufenpyrad_2		10.58	318.1	131.1	14
Tebuthiuron_1	Urea herbicide	5.73	156	62	15
Tebuthiuron_2		5.73	156	74	15
Tefluthrin_1	Pyrethroid insecticide	7.42	177	127	14
Tefluthrin_2		7.42	177	137	16
Terbufos_1	Organophosphorus insecticide and nematicide	7.24	186	97	15
Terbufos_2		7.24	230.9	185	5
terbumeton_1	1,3,5-triazine herbicide	7.13	210.1	100.1	15
terbumeton_2		7.13	225.1	154.1	15
Terbuthylazine_1	1,3,5-triazine herbicide	7.22	214.1	104	16
Terbuthylazine_2		7.22	214.1	132	10
Terbutryn_1	1,3,5-triazine herbicide	8.1	241.1	170.1	12
Terbutryn_2		8.1	241.1	185	6
Tetraconazole_1	Triazole fungicide	8.41	100.9	51	10

Tetraconazole_2		8.41	336	204	28
Tetramethrin_1	Pyrethroid insecticide	10.43	164	77.1	22
Tetramethrin_2		10.43	164	107.1	12
Thiobencarb_1	Thiocarbamate herbicide	8.26	256.9	72	20
Thiobencarb_2		8.26	256.9	100.1	5
Tolclofos-methyl_1	Organophosphate fungicide	7.92	265	219.9	20
Tolclofos-methyl-2		7.92	265	250	12
Tolfenpyrad_1	Pyrazole insecticide	13.17	383.1	145.1	10
Tolfenpyrad_2		13.17	383.1	171.1	20
Triadimefon_1	Triazole fungicide	8.39	208	126.7	12
Triadimefon_2		8.39	208	180.8	8
Triadimenol_1	Triazole fungicide	8.8	128	65	18
Triadimenol_2		8.8	168.2	70	10
Triazophos_1	Organophosphorus insecticide, acaricide, and nematicide	9.83	161	106.1	12
Triazophos_2		9.83	161	134.1	8
Triclopyr-2-butoxyethyl_1	Pyridine herbicide	9.39	181.9	145.9	15
Triclopyr-2-butoxyethyl_2		9.39	211.9	109.9	30
Trifloxystrobin_1	Strobilurin fungicide	9.96	116.1	63	24
Trifloxystrobin_2		9.96	116.1	89	14
Triflumizole_1	Imidazole fungicide	8.86	206	179	14
Triflumizole_2		8.86	206	186	8
Trifluralin_1	Dinitroaniline herbicide	6.62	306.1	206	10
Trifluralin_2		6.62	306.1	264.1	8
Triticonazole_1	Triazole fungicide	10.83	217	167	18
Triticonazole_2		10.83	235.1	181.9	12

Table S3: Limits of quantification (LOQ), linearity (R^2), average recoveries (Rec.), and relative standard deviations (RSD) for 430 pesticides analyzed in onions.

Compound Name	LOQ (mg/kg)	R^2	0.005 ppm		0.01 ppm		0.1 ppm	
			mean %	RSD %	mean %	RSD %	mean %	RSD %
3-hydroxy carbofuran	0.005	0.9991	105%	4.67%	92%	9.56%	85%	5.02%
Abamectin	0.005	0.9985	96%	9.53%	95%	13.39%	91%	14.44%
Acephate	0.005	0.9968	100%	5.25%	100%	4.23%	82%	6.34%
Acequinocyl	0.005	0.9999	94%	10.58%	103%	5.45%	104%	14.63%
Acetamiprid	0.005	0.9767	104%	5.46%	100%	5.65%	94%	5.90%
Acetochlor	0.005	0.9863	83%	4.24%	99%	5.81%	101%	12.97%
Afidopyropen	0.005	0.9985	104%	6.90%	105%	5.59%	106%	6.28%
Alachlor	0.005	0.9978	102%	4.24%	99%	8.32%	100%	3.67%
Aldicarb	0.005	0.9993	101%	4.34%	101%	8.04%	98%	2.00%
Aldicarb Sulfoxide	0.005	0.9892	87%	4.07%	96%	5.02%	96%	5.00%
Aldicarb Sulphone	0.005	0.9979	99%	5.98%	102%	4.44%	100%	4.07%
Aldoxycarb	0.005	0.9985	102%	6.18%	101%	6.34%	100%	4.16%
Aldrin	0.005	0.9985	102%	8.28%	103%	4.63%	99%	3.65%
Alpha cypermethrin	0.005	0.9967	92%	4.47%	102%	5.90%	94%	11.65%
Ametryn	0.005	0.9889	102%	4.07%	98%	12.97%	99%	6.32%
Amicarbazone	0.005	0.9982	104%	4.72%	102%	6.28%	96%	4.80%
Amidosulfuron	0.005	0.9989	94%	4.36%	97%	3.67%	90%	17.39%
Aminocarb	0.005	0.9963	95%	8.11%	98%	5.96%	108%	5.46%
Amisulbrom	0.005	0.9895	108%	7.67%	95%	6.10%	98%	3.38%
Amitraz	0.005	0.9953	103%	4.54%	88%	4.66%	96%	3.05%
Amitrol	0.005	0.9996	106%	5.89%	94%	3.83%	91%	3.61%
Anilofos	0.005	0.9832	107%	3.75%	100%	4.81%	93%	11.47%
Atrazine	0.005	0.9856	109%	5.66%	94%	12.05%	99%	12.68%
Azaconazol	0.005	0.9928	106%	4.70%	91%	14.03%	98%	3.88%
Azamethiphos	0.005	0.9934	89%	6.61%	89%	4.67%	99%	12.96%
Azimsulfuron	0.005	0.9981	86%	7.09%	93%	3.69%	89%	3.09%
Azinophos-Et	0.005	0.9961	94%	11.29%	104%	5.15%	97%	3.44%
Azinphos-Me	0.005	0.9977	96%	11.77%	90%	2.47%	93%	4.14%
Azoxystrobin	0.005	0.9962	92%	4.23%	96%	3.72%	96%	5.36%
Barban	0.005	0.9836	98%	12.48%	87%	4.04%	86%	4.45%
Beflubutamid	0.005	0.9978	95%	11.70%	101%	4.47%	94%	3.58%
Benalaxyl	0.005	0.9892	94%	6.09%	102%	5.15%	87%	5.27%
Bendiocarb	0.005	0.9958	95%	16.34%	97%	4.33%	96%	3.50%
Benomyl (as carbendazim)	0.005	0.9968	103%	3.02%	96%	3.76%	97%	13.22%
Bensulfuron-Me	0.005	0.9939	97%	9.80%	101%	2.88%	92%	2.41%
bentazon	0.005	0.9891	102%	7.67%	98%	5.66%	96%	3.37%
Benthivalicarb isopropyl	0.005	0.9931	91%	11.58%	95%	5.23%	94%	5.16%

Benzoximate	0.005	0.9961	90%	17.17%	84%	6.30%	80%	6.10%
Bifenazate	0.005	0.9962	91%	7.98%	101%	4.73%	102%	14.98%
Bifenrhtin	0.005	0.9923	92%	7.61%	90%	4.01%	100%	3.27%
Biphenyl	0.005	0.9868	91%	17.56%	92%	16.59%	91%	14.79%
Bispyribac	0.005	0.9938	100%	6.24%	100%	6.24%	100%	7.61%
Bitertanol	0.005	0.9936	101%	6.35%	101%	6.35%	103%	4.45%
Boscalid	0.005	0.9968	90%	7.65%	95%	12.47%	96%	10.29%
Bromacil	0.005	0.9976	92%	14.21%	93%	12.53%	91%	9.85%
Bromadiolone	0.005	0.9982	91%	4.23%	91%	4.54%	91%	14.95%
Bromoxynil	0.005	0.9969	98%	7.84%	92%	6.32%	93%	5.71%
Bromuconazole	0.005	0.9929	89%	4.35%	92%	4.98%	93%	4.67%
Bupirimate	0.005	0.9983	100%	3.82%	100%	2.84%	100%	2.80%
Buprofezine	0.005	0.9824	96%	2.66%	99%	4.16%	88%	4.38%
Butocarboxim	0.005	0.9944	107%	12.56%	97%	5.61%	97%	5.96%
Butralin	0.005	0.9936	101%	6.54%	97%	3.31%	98%	3.78%
Cadusafos	0.005	0.9991	88%	4.08%	104%	1.92%	104%	11.97%
Captan	0.005	0.9898	87%	3.62%	95%	4.40%	105%	3.50%
Carbaryl	0.005	0.9974	100%	13.20%	100%	6.48%	99%	6.58%
Carbetamide	0.005	0.9829	90%	9.32%	100%	4.15%	101%	13.36%
Carbofuran	0.005	0.9985	90%	4.84%	108%	4.24%	97%	3.43%
Carbosulfan	0.005	0.9978	88%	14.54%	98%	2.86%	98%	11.75%
Carboxin	0.005	0.9996	99%	16.20%	87%	15.78%	84%	13.27%
Carfentrazone ethyl	0.005	0.9991	94%	7.17%	95%	9.94%	97%	10.04%
Chlorantraniliprole	0.005	0.9964	92%	8.98%	99%	6.32%	99%	4.23%
Chlorbromuron	0.005	0.9952	104%	12.97%	90%	7.42%	91%	7.84%
Chlordane-cis	0.005	0.9937	90%	8.53%	89%	4.64%	82%	4.35%
Chlordane-trans	0.005	0.9985	97%	13.67%	103%	3.74%	101%	3.82%
Chlorfenapyr	0.005	0.9939	105%	9.78%	104%	3.75%	104%	12.66%
Chlorfenvinphos	0.005	0.9978	88%	12.06%	96%	4.02%	96%	2.56%
Chlorfluazuron	0.005	0.9968	93%	11.35%	101%	7.68%	93%	6.54%
Chloridazon	0.005	0.9936	90%	11.86%	99%	4.44%	104%	4.08%
Chlorothalonil	0.005	0.9959	90%	6.47%	98%	3.84%	97%	3.62%
Chlorotoluron	0.005	0.9952	99%	8.90%	85%	8.28%	84%	8.51%
Chloroxuron	0.005	0.9983	86%	9.69%	80%	7.19%	80%	6.79%
Chlorpropham	0.005	0.9986	94%	6.09%	102%	5.15%	87%	5.27%
Chlorpyrifos	0.005	0.9993	95%	16.34%	97%	4.33%	96%	3.50%
Chlorpyrifos-Me	0.005	0.9905	103%	3.02%	96%	3.76%	97%	13.22%
chlorsulfuron	0.005	0.9955	97%	9.80%	101%	2.88%	92%	2.41%
Chlorthiophose	0.005	0.9988	102%	7.67%	98%	5.66%	96%	3.37%
Chlortoluron	0.005	0.9939	91%	11.58%	95%	5.23%	94%	5.16%
Chromofenozide	0.005	0.9899	90%	17.17%	84%	6.30%	80%	6.10%

Cinidon-Ethyl	0.005	0.9967	91%	7.98%	101%	4.73%	102%	14.98%
Cinosulfuron	0.005	0.9961	92%	7.61%	90%	4.01%	100%	3.27%
Clethodim	0.005	0.9899	91%	17.56%	92%	16.59%	91%	14.79%
Clodinafop-propargy	0.005	0.9867	100%	6.24%	100%	6.24%	100%	7.61%
Clofentezine	0.005	0.9963	101%	6.35%	101%	6.35%	103%	4.45%
Clomazone	0.005	0.9987	90%	7.65%	95%	12.47%	96%	10.29%
Clopyralid	0.005	0.9927	92%	14.21%	93%	12.53%	91%	9.85%
Clothianidin	0.005	0.9903	91%	4.23%	91%	4.54%	91%	14.95%
Coumaphos	0.005	0.9983	98%	7.84%	92%	6.32%	93%	5.71%
Coumatetray	0.005	0.9897	89%	4.35%	92%	4.98%	93%	4.67%
Cyanophos	0.005	0.9976	100%	3.82%	100%	2.84%	100%	2.80%
Cyantraniliprole	0.005	0.9961	96%	2.66%	99%	4.16%	88%	4.38%
Cyazofamid	0.005	0.9809	107%	12.56%	97%	5.61%	97%	5.96%
Cycloxydim	0.005	0.9919	101%	6.54%	97%	3.31%	98%	3.78%
Cyflufenamid	0.005	0.9989	88%	4.08%	104%	1.92%	104%	11.97%
Cyflumetofen	0.005	0.9963	87%	3.62%	95%	4.40%	105%	3.50%
Cyfluthrin	0.005	0.9982	86%	8.51%	93%	3.97%	94%	12.69%
Cyhalofop-butyl	0.005	0.9949	86%	6.79%	94%	4.82%	92%	4.71%
Cyhalothrin-gamma	0.005	0.9999	76%	5.27%	85%	17.23%	86%	15.29%
Cymoxanil	0.005	0.9967	86%	3.50%	96%	5.37%	97%	5.23%
Cyphenothrin	0.005	0.9963	92%	3.22%	78%	5.20%	88%	5.34%
Cypermethrin	0.005	0.9894	96%	2.41%	103%	3.32%	102%	3.42%
Cyproconazole	0.005	0.9901	95%	3.37%	99%	4.66%	99%	4.94%
Cyprodinil	0.005	0.9971	100%	5.16%	93%	4.79%	92%	13.90%
Cyromazin	0.005	0.9983	92%	6.10%	78%	3.59%	78%	3.48%
Dazomet	0.005	0.9987	95%	9.87%	76%	4.94%	75%	5.24%
DDD-o,p`	0.005	0.9927	79%	14.68%	82%	4.90%	103%	12.63%
DDD-p,p`	0.005	0.9963	92%	12.21%	79%	5.19%	109%	4.58%
DDE-p,p`	0.005	0.9987	80%	6.48%	87%	4.50%	96%	4.61%
DDT-o,p`	0.005	0.9927	76%	7.37%	81%	3.79%	102%	3.38%
DDT-p,p`	0.005	0.9963	86%	12.83%	90%	9.23%	99%	12.25%
Deltamethrin	0.005	0.9903	98%	14.66%	103%	3.27%	103%	2.71%
Demeton-S-methyl	0.005	0.9973	101%	4.79%	95%	4.54%	91%	4.95%
Desmedipham	0.005	0.9995	85%	7.58%	98%	5.12%	98%	3.99%
Diafenthiuron	0.005	0.9989	92%	6.07%	91%	4.88%	90%	4.59%
Diazinon	0.005	0.9963	89%	15.37%	98%	6.26%	99%	4.68%
Dichlofenthion	0.005	0.9929	93%	13.11%	101%	3.17%	103%	2.60%
Dichlofuanid	0.005	0.9909	96%	5.35%	94%	8.97%	92%	5.63%
Diclofop methyl	0.005	0.9958	108%	3.52%	94%	10.39%	94%	11.38%
Dichlorvos	0.005	0.9896	97%	10.28%	92%	6.42%	91%	6.56%
Dicotophos	0.005	0.9939	90%	3.55%	100%	3.41%	99%	2.56%

Dieldrin	0.005	0.9982	87%	8.28%	101%	5.12%	101%	5.18%
Diethofencarb	0.005	0.9896	92%	13.22%	103%	3.33%	103%	3.14%
Difenoconazole	0.005	0.9879	96%	8.47%	82%	8.59%	83%	8.12%
Difenthiuron	0.005	0.9985	96%	9.55%	95%	9.05%	97%	8.66%
Diflubenzuron	0.005	0.9985	95%	14.04%	101%	8.07%	92%	7.77%
Diflufenican	0.005	0.9967	96%	12.90%	89%	3.75%	82%	13.82%
Dimetaclor	0.005	0.9879	95%	13.73%	105%	3.33%	105%	2.64%
Dimethenamide	0.005	0.9905	93%	11.10%	103%	3.03%	103%	2.48%
Dimethoate	0.005	0.9985	91%	5.02%	94%	6.54%	92%	5.64%
Dimethomorph	0.005	0.9993	95%	7.03%	104%	7.93%	102%	5.52%
Diniconazole	0.005	0.9931	112%	5.54%	104%	3.48%	103%	3.13%
Dinotefuran	0.005	0.9923	96%	5.30%	106%	2.45%	106%	2.44%
Diphenylamine	0.005	0.9955	89%	14.73%	90%	2.99%	91%	12.35%
Diphacinone	0.005	0.9968	84%	12.39%	92%	7.07%	91%	7.57%
Disulfoton	0.005	0.9929	97%	5.63%	76%	5.01%	76%	5.49%
Diuron	0.005	0.9989	97%	9.72%	96%	5.79%	94%	4.56%
Dodemorph	0.005	0.9963	92%	10.08%	65%	3.85%	65%	4.30%
Dodine	0.005	0.9885	86%	8.60%	86%	8.60%	86%	8.58%
Edifenophos	0.005	0.986	94%	9.75%	69%	4.30%	89%	3.61%
Emamectin	0.005	0.9982	100%	11.60%	102%	11.98%	102%	11.19%
Endosulfan-alpha	0.005	0.9961	82%	8.75%	96%	4.79%	95%	4.30%
Endosulfan-beta	0.005	0.9981	84%	13.99%	93%	7.94%	93%	5.42%
Endosulfan-sulphate	0.005	0.9868	81%	4.47%	100%	3.36%	100%	2.44%
Endrin	0.005	0.9898	80%	4.96%	100%	4.29%	100%	3.10%
EPN	0.005	0.9864	89%	13.24%	84%	5.88%	83%	4.14%
Epoxiconazole	0.005	0.9929	90%	6.35%	88%	4.79%	88%	5.29%
Eprinomectin	0.005	0.9989	92%	12.47%	77%	5.21%	77%	2.79%
Esfenvalerate	0.005	0.9963	103%	12.53%	94%	6.50%	92%	4.28%
Ethiofencarb	0.005	0.9786	104%	4.54%	91%	4.92%	91%	5.78%
Ethiofencarb Sulfon	0.005	0.9968	89%	6.32%	77%	4.11%	77%	4.47%
Ethiofencarb Sulfoxid	0.005	0.9938	94%	4.98%	103%	7.85%	105%	8.96%
Ethion	0.005	0.9856	86%	2.84%	91%	2.84%	102%	1.99%
Ethiprole	0.005	0.9924	103%	4.16%	100%	5.17%	99%	3.01%
Ethirimol	0.005	0.9997	99%	5.61%	98%	13.12%	99%	12.17%
Ethofumesate	0.005	0.9769	95%	3.31%	89%	6.74%	89%	8.11%
Ethoprophos	0.005	0.9985	98%	1.92%	100%	2.71%	100%	2.36%
Ethulenthiourea	0.005	0.9769	101%	4.40%	89%	3.93%	89%	2.74%
Etofenprox	0.005	0.9964	91%	3.97%	95%	6.31%	94%	6.85%
Etoxazole	0.005	0.9987	106%	4.82%	77%	3.85%	77%	3.70%
Etrimfos	0.005	0.9879	91%	17.23%	92%	8.01%	91%	6.30%
Famoxadone	0.005	0.9962	99%	5.37%	94%	10.07%	93%	11.29%

Fenamidone	0.005	0.9988	107%	5.20%	89%	7.88%	88%	6.90%
Fenamiphos	0.005	0.9971	102%	3.32%	97%	6.24%	96%	1.07%
Fenarimol	0.005	0.9917	98%	14.66%	103%	3.27%	103%	2.71%
Fenazaquin	0.005	0.9982	101%	4.79%	95%	4.54%	91%	4.95%
Fenbuconazole	0.005	0.9969	85%	7.58%	98%	5.12%	98%	3.99%
Fenbutatin oxide	0.005	0.9946	92%	6.07%	91%	4.88%	90%	4.59%
Fenhexamid	0.005	0.9978	89%	15.37%	98%	6.26%	99%	4.68%
Fenitrothion	0.005	0.9988	93%	13.11%	101%	3.17%	103%	2.60%
Fenoxap-p-ethyl	0.005	0.9971	96%	5.35%	94%	8.97%	92%	5.63%
Fenoxycarb	0.005	0.9955	108%	3.52%	94%	10.39%	94%	11.38%
Fenpropathrin	0.005	0.9968	97%	10.28%	92%	6.42%	91%	6.56%
Fenpropidin	0.005	0.9795	90%	3.55%	100%	3.41%	99%	2.56%
Fenpropimorph	0.005	0.988	87%	8.28%	101%	5.12%	101%	5.18%
Fenpyroxazamine	0.005	0.9968	92%	13.22%	103%	3.33%	103%	3.14%
Fenpyroximate	0.005	0.9875	96%	8.47%	82%	8.59%	83%	8.12%
Fenthion	0.005	0.9968	96%	9.55%	95%	9.05%	97%	8.66%
Fenvalerat	0.005	0.9896	95%	14.04%	101%	8.07%	92%	7.77%
Fipronil	0.005	0.9983	96%	12.90%	89%	3.75%	82%	13.82%
Flonicamid	0.005	0.9925	95%	13.73%	105%	3.33%	105%	2.64%
Florasulam	0.005	0.9975	93%	11.10%	103%	3.03%	103%	2.48%
Fluazifop-p-butyl	0.005	0.9769	91%	5.02%	94%	6.54%	92%	5.64%
Fluazinam	0.005	0.9964	95%	7.03%	104%	7.93%	102%	5.52%
Flubendiamide	0.005	0.9987	112%	5.54%	104%	3.48%	103%	3.13%
Flucythrinate	0.005	0.9879	96%	5.30%	106%	2.45%	106%	2.44%
Fludioxonil	0.005	0.9989	89%	14.73%	90%	2.99%	91%	12.35%
Flufenacet	0.005	0.9689	89%	6.00%	102%	6.35%	104%	4.87%
Flufenoxuron	0.005	0.9953	88%	11.25%	103%	5.03%	94%	5.01%
Flumetesulam	0.005	0.9873	84%	10.19%	96%	2.97%	95%	2.55%
Flumeturon	0.005	0.9809	91%	10.87%	97%	4.77%	107%	13.93%
Flumioxazin	0.005	0.9983	90%	9.71%	100%	5.06%	99%	4.92%
Fluopicolide	0.005	0.9965	92%	8.99%	90%	5.90%	90%	6.47%
Fluopyram	0.005	0.9805	103%	7.31%	91%	7.37%	92%	15.42%
Flupyradifurone	0.005	0.9965	99%	7.17%	104%	6.34%	104%	4.47%
Fluquinconazole	0.005	0.9962	89%	4.97%	96%	4.02%	106%	4.42%
Fluroxypyr	0.005	0.9808	95%	9.83%	99%	7.51%	101%	5.84%
Fluroxypyr-meptyl	0.005	0.9886	99%	3.36%	95%	3.07%	94%	12.04%
Flusilazole	0.005	0.9782	99%	7.06%	101%	3.87%	100%	3.35%
Flutamone	0.005	0.9877	91%	14.88%	100%	3.38%	101%	2.52%
Flutolanil	0.005	0.9968	92%	19.01%	100%	2.93%	101%	2.98%
Flutriafol	0.005	0.9952	96%	5.78%	98%	2.74%	98%	11.83%
Fluxapyroxad	0.005	0.9769	95%	5.60%	98%	4.98%	98%	4.28%

Folpet	0.005	0.9964	97%	9.70%	94%	2.80%	93%	11.85%
Fomesafen	0.005	0.9987	103%	8.63%	77%	4.67%	78%	3.21%
Foramsulfuron	0.005	0.9879	103%	3.29%	104%	5.56%	85%	5.31%
Formetanate	0.005	9958	96%	4.32%	83%	21.90%	80%	17.97%
Fosthiazate	0.005	0.9921	89%	9.28%	100%	3.62%	101%	3.28%
Halauxifen	0.005	0.9967	97%	6.58%	98%	3.84%	99%	3.10%
Halauxifen-methyl	0.005	0.9962	87%	9.89%	97%	6.05%	96%	12.69%
Halosulfuron-methyl	0.005	0.9867	98%	8.21%	92%	11.47%	91%	9.62%
Haloxyfop Methyl	0.005	0.9877	92%	5.01%	100%	5.18%	100%	4.85%
HCH-alpha	0.005	0.9986	91%	4.07%	102%	3.09%	91%	12.92%
HCH-beta	0.005	0.9974	91%	7.57%	101%	3.53%	100%	12.99%
HCH-delta	0.005	0.9992	85%	7.99%	98%	3.77%	98%	3.04%
HCH-gamma (lindane)	0.005	0.9989	96%	6.57%	103%	3.24%	103%	3.22%
Heptachlor	0.005	0.9857	98%	4.02%	98%	6.41%	97%	6.38%
Heptachlor-endo-epoxide	0.005	0.9871	93%	3.13%	98%	7.26%	97%	5.81%
Heptachlor-exo-epoxide	0.005	0.9791	88%	3.71%	94%	3.74%	94%	3.04%
Hexachlorobenzene	0.005	0.9877	83%	3.60%	94%	3.78%	94%	2.98%
Hexaflumeron	0.005	0.9929	97%	7.57%	100%	3.61%	101%	2.50%
Hexazinone	0.005	0.9769	90%	5.49%	98%	3.61%	97%	2.98%
Hexythiazox	0.005	0.9964	93%	4.56%	95%	3.94%	95%	3.94%
Hymexazol	0.005	0.9987	88%	4.30%	103%	9.51%	104%	6.79%
Imazalil	0.005	0.9879	94%	8.58%	97%	11.56%	87%	12.20%
Imazamethabenz-Me	0.005	0.9985	96%	3.61%	97%	4.03%	97%	3.40%
Imazamox	0.005	0.9954	102%	11.19%	106%	8.75%	109%	4.86%
Imazapic	0.005	0.9857	97%	4.30%	104%	2.53%	94%	2.10%
Imazapyr	0.005	0.9769	94%	5.42%	104%	13.33%	101%	7.50%
Imazaquin	0.005	0.9876	95%	2.44%	99%	3.13%	100%	11.93%
Imicyafos	0.005	0.9958	90%	3.10%	92%	1.48%	92%	11.13%
Imidacloprid	0.005	0.9847	92%	4.14%	87%	10.38%	87%	8.61%
Iminoctadine	0.005	0.9992	94%	5.29%	94%	7.71%	96%	7.45%
Indaziflam	0.005	0.9877	93%	2.79%	98%	2.62%	98%	12.87%
Indoxacarb	0.005	0.9982	96%	7.24%	106%	6.67%	108%	3.80%
Iodosulfuron-Me	0.005	0.9962	98%	12.66%	94%	4.22%	104%	4.02%
Iprobenfos	0.005	0.9878	99%	8.81%	99%	4.23%	100%	4.72%
Iprodione	0.005	0.9985	94%	4.25%	100%	6.53%	102%	6.66%
Iprovalicarb	0.005	0.9906	99%	3.36%	95%	3.07%	94%	12.04%
Isoprothiolane	0.005	0.9861	99%	7.06%	101%	3.87%	100%	3.35%
Isoproturon	0.005	0.9758	91%	14.88%	100%	3.38%	101%	2.52%
Isoxaflutole	0.005	0.9877	92%	19.01%	100%	2.93%	101%	2.98%
Kresoxim-methyl	0.005	0.9986	96%	5.78%	98%	2.74%	98%	11.83%
Lambda-Cyhalothrin	0.005	0.9989	95%	5.60%	98%	4.98%	98%	4.28%

Lenacil	0.005	0.9968	97%	9.70%	94%	2.80%	93%	11.85%
Linuron	0.005	0.9879	103%	8.63%	77%	4.67%	78%	3.21%
Lufenuron	0.005	0.9867	103%	3.29%	104%	5.56%	85%	5.31%
Malaoxon	0.005	0.9928	96%	4.32%	83%	21.90%	80%	17.97%
Malathion	0.005	0.9877	89%	9.28%	100%	3.62%	101%	3.28%
Mandipropamid	0.005	0.9987	97%	6.58%	98%	3.84%	99%	3.10%
MCPA	0.005	0.9799	87%	9.89%	97%	6.05%	96%	12.69%
Mecarbam	0.005	0.9983	98%	8.21%	92%	11.47%	91%	9.62%
Mefenacet	0.005	0.9988	92%	5.01%	100%	5.18%	100%	4.85%
Mefentrifluconazole	0.005	0.9985	91%	4.07%	102%	3.09%	91%	12.92%
Mefenoxam	0.005	0.9979	91%	7.57%	101%	3.53%	100%	12.99%
Mefenpyr-diethyl	0.005	0.9974	85%	7.99%	98%	3.77%	98%	3.04%
Melbmectin	0.005	0.9985	96%	6.57%	103%	3.24%	103%	3.22%
Meolachlor	0.005	0.9976	98%	4.02%	98%	6.41%	97%	6.38%
Mepanipyrim	0.005	0.9982	93%	3.13%	98%	7.26%	97%	5.81%
Mepronil	0.005	0.9986	88%	3.71%	94%	3.74%	94%	3.04%
Mesosulfuron-Me	0.005	0.9897	99%	5.62%	98%	3.66%	98%	13.02%
Mesotrione	0.005	0.9979	78%	5.52%	87%	11.56%	87%	12.20%
Metaflumizone	0.005	0.9809	89%	3.98%	73%	4.79%	72%	3.90%
Metalaxyl	0.005	0.9986	91%	3.19%	95%	4.55%	95%	13.12%
Metalaxyl-M	0.005	0.9976	93%	2.80%	96%	4.55%	97%	3.46%
Metamitron	0.005	0.9874	82%	7.45%	79%	5.06%	83%	4.99%
Metazachlor	0.005	0.9983	102%	3.28%	105%	6.14%	105%	7.42%
Metconazole	0.005	0.9868	94%	13.36%	103%	5.49%	105%	3.53%
Methabenzthiazuron	0.005	0.9978	92%	7.64%	99%	3.69%	99%	3.24%
Methacrifos	0.005	0.9978	101%	5.78%	90%	5.94%	101%	6.26%
Methamidophos	0.005	0.9988	93%	15.01%	101%	3.06%	100%	2.81%
Methidathion	0.005	0.9986	99%	11.11%	99%	3.60%	99%	3.55%
Methiocarb	0.005	0.9978	98%	11.35%	98%	5.94%	100%	4.76%
Methiocarb Sulfoxid	0.005	0.9985	91%	9.55%	87%	4.04%	87%	4.45%
Methiocarb Sulphone	0.005	0.9977	91%	11.34%	97%	2.98%	97%	12.51%
Methomyl	0.005	0.9988	96%	11.13%	99%	3.28%	89%	12.61%
Methoxychlor	0.005	0.9983	91%	2.52%	94%	3.59%	95%	2.38%
Methoxyfenozide	0.005	0.9874	92%	9.98%	99%	7.81%	97%	8.50%
Metobromuron	0.005	0.9879	96%	11.83%	94%	4.20%	94%	3.87%
Metoxuron	0.005	0.9876	100%	4.28%	94%	3.22%	94%	2.14%
Metrafenone	0.005	0.9968	99%	1.85%	98%	2.86%	98%	11.75%
Metribuzin	0.005	0.9989	83%	3.21%	103%	3.32%	102%	3.42%
Metsulfuron Methyl	0.005	0.9968	105%	5.31%	101%	3.79%	102%	3.38%
Mevenphos	0.005	0.9938	93%	17.97%	105%	3.83%	105%	2.75%
Monocrotophos	0.005	0.9869	98%	3.28%	96%	3.64%	94%	2.73%

Monolinuron	0.005	0.9985	90%	3.10%	90%	3.96%	90%	3.04%
Monuron	0.005	0.9979	87%	2.69%	96%	5.79%	94%	4.56%
Moxidectin	0.005	0.9963	94%	9.62%	97%	6.24%	96%	1.07%
Myclobutanil	0.005	0.9985	99%	4.85%	90%	5.04%	101%	4.48%
Napropamide	0.005	0.9979	96%	2.92%	104%	7.93%	102%	5.52%
Nicosulfuron	0.005	0.9958	90%	2.99%	98%	5.14%	99%	5.46%
Nitenpyram	0.005	0.9874	98%	3.04%	96%	2.97%	95%	12.55%
Novaluron	0.005	0.9886	101%	12.58%	88%	6.43%	88%	5.29%
Nuarimol	0.005	0.9967	94%	8.60%	99%	4.66%	99%	4.94%
Ofurace	0.005	0.9901	97%	9.24%	92%	6.62%	92%	6.59%
Omethoate	0.005	0.9836	92%	6.17%	94%	3.47%	94%	12.28%
Ortho-phenylphenol	0.005	0.9976	95%	7.84%	95%	6.21%	96%	7.09%
Orthosulfamuron	0.005	0.9877	98%	9.74%	85%	5.46%	85%	4.57%
Oxadiargyl	0.005	0.9986	93%	8.08%	100%	2.72%	99%	12.26%
Oxadiazon	0.005	0.9989	97%	10.35%	93%	5.15%	95%	3.86%
Oxadixyl	0.005	0.9968	97%	9.30%	97%	5.71%	97%	5.12%
Oxamyl	0.005	0.9879	90%	12.22%	95%	4.58%	104%	4.52%
Oxasulfuron	0.005	0.9968	99%	7.54%	89%	2.54%	99%	0.92%
Oxycarboxin	0.005	0.9936	88%	10.07%	95%	4.09%	96%	3.36%
Oxyflurfen	0.005	0.9959	96%	5.18%	96%	3.46%	96%	12.31%
Paraoxon Methyl	0.005	0.9955	99%	4.66%	97%	2.86%	97%	3.10%
Parathion Ethyl	0.005	0.9983	89%	10.42%	85%	4.84%	84%	4.60%
Penconazole	0.005	0.9986	88%	7.32%	96%	3.79%	97%	3.23%
Pencycuron	0.005	0.9997	91%	6.19%	100%	3.25%	100%	3.01%
Pendimethalin	0.005	0.9905	89%	12.89%	90%	5.28%	90%	4.61%
penflufen	0.005	0.9955	104%	6.81%	103%	6.08%	101%	4.42%
Penoxulam	0.005	0.9988	97%	10.66%	104%	7.03%	85%	6.93%
Penthiopyrad	0.005	0.9939	92%	12.88%	104%	7.95%	103%	3.66%
Permethrin	0.005	0.9899	99%	9.89%	96%	3.00%	96%	2.58%
Phenmedipham	0.005	0.9967	89%	6.03%	100%	2.58%	101%	11.97%
Phenthoate	0.005	0.9961	102%	13.28%	102%	3.98%	101%	3.53%
Phorate	0.005	0.9899	107%	6.52%	95%	4.45%	84%	4.00%
Phosalone	0.005	0.9867	93%	12.63%	97%	6.70%	98%	5.02%
Phosmet	0.005	0.9879	100%	17.16%	82%	5.23%	84%	12.32%
Phosphamidon	0.005	0.9985	96%	13.08%	101%	4.05%	101%	3.73%
Phoxim	0.005	0.9954	92%	10.47%	95%	7.82%	93%	3.72%
Picarbutrazox	0.005	0.9857	99%	11.21%	104%	5.10%	105%	5.10%
Picolinafen	0.005	0.9769	90%	12.56%	102%	6.71%	102%	7.96%
Picoxystrobin	0.005	0.9876	90%	5.07%	99%	7.06%	98%	7.76%
pinoxaden	0.005	0.9958	96%	2.55%	93%	3.29%	103%	3.10%
Piperonyl butoxid	0.005	0.9887	89%	13.78%	94%	2.62%	94%	11.23%

Pirimicarb	0.005	0.9992	88%	11.34%	97%	6.78%	98%	6.83%
Pirimicarb desmethyl	0.005	0.9877	108%	9.30%	96%	5.03%	97%	5.14%
Pirimiphos Ethyl	0.005	0.9982	89%	7.03%	94%	4.25%	94%	3.73%
Pirimiphos Methyl	0.005	0.9962	90%	7.15%	95%	2.96%	87%	2.39%
praziquantel	0.005	0.9978	90%	15.58%	92%	3.18%	92%	2.78%
pretilachlor	0.005	0.9985	93%	8.16%	99%	3.36%	99%	2.60%
Prochloraz	0.005	0.9906	87%	8.07%	109%	6.62%	99%	6.65%
Prodiamine	0.005	0.9867	95%	10.60%	93%	10.43%	95%	4.90%
Profenofos	0.005	0.9758	101%	7.69%	89%	5.92%	89%	6.33%
Promecarb	0.005	0.9877	97%	17.57%	93%	4.17%	93%	3.90%
Prometryn	0.005	0.9986	92%	16.17%	96%	4.32%	95%	3.90%
Propachlor	0.005	0.9808	86%	10.03%	101%	9.06%	98%	7.10%
Propamocarb	0.005	0.9886	93%	11.38%	89%	5.65%	89%	3.92%
Propanil	0.005	0.9782	78%	7.26%	101%	5.96%	100%	4.83%
Propaquizafop	0.005	0.9877	87%	10.33%	97%	2.95%	97%	2.50%
Propargite	0.005	0.9968	95%	9.02%	89%	2.99%	99%	2.77%
Propazine	0.005	0.9952	87%	10.10%	99%	3.79%	99%	2.69%
Propetamphos	0.005	0.9769	96%	8.45%	101%	3.99%	101%	3.74%
Propiconazole	0.005	0.9964	72%	17.86%	99%	4.88%	99%	5.24%
Propoxur	0.005	0.9987	99%	8.47%	100%	3.25%	90%	2.84%
Propyzamide	0.005	0.9979	94%	4.08%	102%	5.35%	102%	5.07%
Proquinazid	0.005	0.9957	93%	12.82%	103%	7.00%	106%	5.66%
Prosulfocarb	0.005	0.9921	89%	11.59%	94%	3.91%	95%	2.72%
Prothiofos	0.005	0.9967	89%	8.76%	95%	2.96%	95%	2.79%
Prothioconazole	0.005	0.9962	91%	6.15%	96%	2.81%	97%	3.14%
Pydiflumetofen	0.005	0.9867	88%	10.21%	100%	3.00%	100%	2.11%
Pymetrozine	0.005	0.9977	98%	7.65%	97%	4.62%	97%	4.32%
Pyraclostrbin	0.005	0.9986	94%	13.40%	97%	16.73%	94%	19.73%
Pyraflufen ethyl	0.005	0.9977	94%	5.32%	91%	6.06%	90%	6.57%
Pyrazophos	0.005	0.9992	98%	15.40%	96%	4.26%	96%	3.07%
Pyrazosulfuron Ethyl	0.005	0.9989	93%	8.95%	102%	11.52%	102%	10.93%
Pyrazoxyfen	0.005	0.9857	89%	9.40%	96%	6.25%	95%	4.28%
Pyridaben	0.005	0.9986	94%	13.36%	103%	5.49%	105%	3.53%
Pyridalyl	0.005	0.9993	92%	7.64%	99%	3.69%	99%	3.24%
Pyridaphenthion	0.005	0.9905	101%	5.78%	90%	5.94%	101%	6.26%
Pyrifenox	0.005	0.9955	93%	15.01%	101%	3.06%	100%	2.81%
Pyrimethanil	0.005	0.9988	99%	11.11%	99%	3.60%	99%	3.55%
Pyriproxyfen	0.005	0.9979	98%	11.35%	98%	5.94%	100%	4.76%
Pyroxsulam	0.005	0.9899	91%	9.55%	87%	4.04%	87%	4.45%
Quinalphos	0.005	0.9967	91%	11.34%	97%	2.98%	97%	12.51%
Quinclorac	0.005	0.9961	96%	11.13%	99%	3.28%	89%	12.61%

Quinmerac	0.005	0.9899	91%	2.52%	94%	3.59%	95%	2.38%
Quinoxyfen	0.005	0.9867	92%	9.98%	99%	7.81%	97%	8.50%
Quizalofop Ethyl	0.005	0.9963	96%	11.83%	94%	4.20%	94%	3.87%
Rimsulfuron	0.005	0.9987	100%	4.28%	94%	3.22%	94%	2.14%
Rotenone	0.005	0.9927	99%	1.85%	98%	2.86%	98%	11.75%
Sebuthylazine	0.005	0.9903	83%	3.21%	103%	3.32%	102%	3.42%
Sebuthylazine-desethyl	0.005	0.9983	105%	5.31%	101%	3.79%	102%	3.38%
Simazine	0.005	0.9897	93%	17.97%	105%	3.83%	105%	2.75%
Spinosad	0.005	0.9976	98%	3.28%	96%	3.64%	94%	2.73%
Spinotram	0.005	0.9961	90%	3.10%	90%	3.96%	90%	3.04%
Spirodiclofen	0.005	0.9809	87%	2.69%	96%	5.79%	94%	4.56%
Spiromesifen	0.005	0.9919	94%	9.62%	97%	6.24%	96%	1.07%
Spirotetramate	0.005	0.9989	99%	4.85%	90%	5.04%	101%	4.48%
Spiroxamin	0.005	0.9963	96%	2.92%	104%	7.93%	102%	5.52%
Sulcotrione	0.005	0.9982	90%	2.99%	98%	5.14%	99%	5.46%
Sulfometuron-methyl	0.005	0.9949	98%	3.04%	96%	2.97%	95%	12.55%
Sulfotep	0.005	0.9999	98%	13.22%	95%	5.81%	94%	6.20%
Sulfoxaflor	0.005	0.9977	96%	6.38%	104%	6.34%	104%	4.47%
Sulphur	0.005	0.9983	101%	5.81%	97%	3.04%	96%	3.39%
Sulprofos	0.005	0.9894	93%	3.04%	92%	6.32%	93%	5.71%
Tebuconazole	0.005	0.9863	89%	3.02%	92%	1.48%	92%	11.13%
Tebufenozide	0.005	0.9985	98%	12.20%	97%	2.86%	97%	3.10%
Tebufenpyrad	0.005	0.9978	99%	3.90%	99%	7.06%	88%	7.76%
Tebuthiuron	0.005	0.9993	88%	8.53%	102%	3.18%	92%	2.78%
Teflubenzuron	0.005	0.9892	86%	12.18%	99%	3.79%	99%	2.69%
Tefluthrin	0.005	0.9999	96%	13.21%	91%	6.06%	90%	6.57%
Temephos	0.005	0.9985	98%	6.66%	94%	4.20%	94%	3.87%
TEPP	0.005	0.9985	98%	4.22%	98%	5.14%	99%	5.46%
Tepraloxydim	0.005	0.9967	85%	11.28%	97%	4.77%	97%	4.24%
Terbufos	0.005	0.9989	91%	7.08%	98%	7.82%	100%	6.90%
Terbumeton	0.005	0.9982	90%	13.86%	97%	4.77%	97%	4.24%
Terbuthylazine	0.005	0.9989	104%	5.43%	100%	5.33%	102%	4.34%
Terbutryn	0.005	0.9963	101%	5.96%	97%	5.24%	91%	4.07%
Tetraconazole	0.005	0.9995	104%	6.10%	102%	6.22%	104%	5.45%
Tetramethrin	0.005	0.9953	100%	4.66%	92%	4.97%	89%	5.65%
Thiabendazole	0.005	0.9896	101%	3.83%	102%	3.26%	86%	5.81%
Thiacloprid	0.005	0.9832	102%	4.81%	107%	9.60%	96%	5.59%
Thiamethoxam	0.005	0.9999	93%	12.05%	100%	10.18%	98%	8.32%
Thiencarbazone-methyl	0.005	0.9983	101%	14.03%	90%	3.41%	98%	8.04%
Thifensulfuron Methyl	0.005	0.9988	105%	4.67%	92%	9.56%	85%	5.02%
Thiobencarb	0.005	0.9995	96%	9.53%	95%	13.39%	91%	14.44%

Thiocyclam-OH	0.005	0.9979	106%	2.83%	98%	3.39%	97%	2.76%
Thiodicarb	0.005	0.9974	100%	8.19%	92%	11.16%	97%	3.20%
Thiofanox	0.005	0.9985	96%	2.80%	80%	15.62%	89%	2.46%
Thiophanate Methyl	0.005	0.9976	108%	4.07%	105%	10.94%	87%	7.10%
Thiuram	0.005	0.9982	99%	2.50%	92%	2.32%	100%	4.76%
Tolclophos Methy	0.005	0.9986	95%	3.34%	96%	9.67%	84%	2.79%
Tolfenpyrad	0.005	0.9897	87%	8.34%	95%	3.06%	101%	2.97%
Tolylfluanid	0.005	0.9999	87%	7.04%	89%	12.85%	97%	1.74%
Tralkoxydim	0.005	0.9809	105%	3.37%	90%	3.60%	99%	2.63%
Triadimifon	0.005	0.9986	80%	7.31%	89%	5.29%	101%	3.25%
Triadiminol	0.005	0.9996	80%	5.91%	84%	4.67%	101%	5.08%
Triasulfuron	0.005	0.9894	84%	3.09%	100%	7.83%	99%	10.03%
Triazophos	0.005	0.9983	92%	5.58%	96%	3.24%	94%	10.45%
Trichlorfon	0.005	0.9868	89%	6.98%	99%	3.54%	104%	4.13%
Triclopyr butatyl	0.005	0.9978	95%	9.03%	83%	3.94%	97%	5.35%
Tricyclazole	0.005	0.9998	99%	2.49%	85%	8.30%	84%	2.24%
Trifloxystrobin	0.005	0.9896	84%	3.72%	88%	5.94%	92%	3.61%
Triflumizole	0.005	0.9939	100%	12.61%	83%	2.82%	89%	2.87%
Triflumuron	0.005	0.9982	90%	4.09%	102%	6.85%	95%	11.33%
Trifluralin	0.005	0.9896	96%	2.49%	97%	3.36%	109%	14.94%
Triflusulfuron methyl	0.005	0.9889	95%	4.28%	99%	4.78%	84%	10.78%
Triforine	0.005	0.9995	89%	5.29%	101%	3.83%	90%	2.03%
Triticonazole	0.005	0.9995	97%	4.94%	101%	7.54%	90%	9.89%
Zoxamide	0.005	0.9867	93%	6.59%	102%	9.17%	96%	2.81%

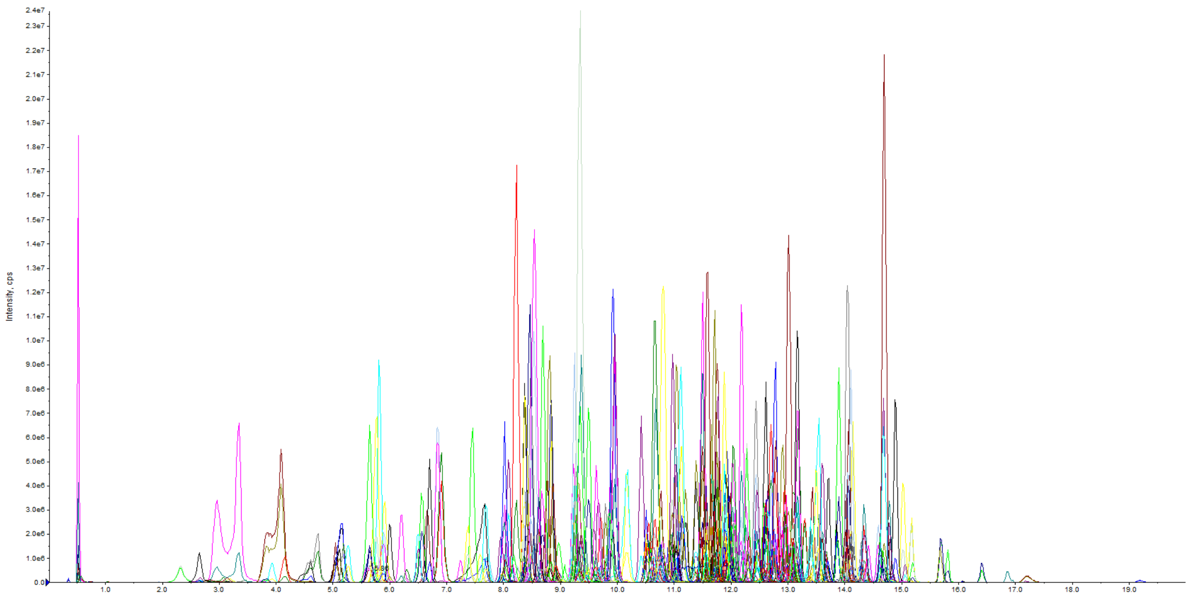

Figure S1: LC-MS/MS chromatogram of analytical standard of LC analytes at 0.1 mg/kg.

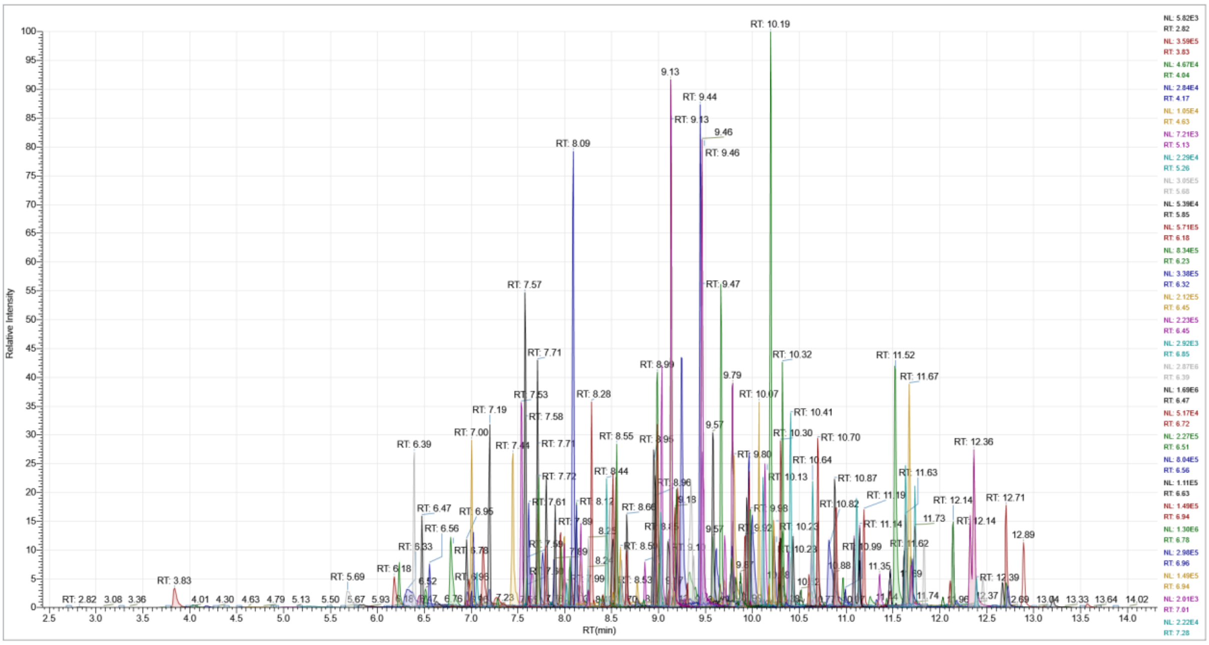

Figure S2: GC-MS/MS chromatogram of analytical standard of GC analytes at 0.1 mg/kg.
